# Maximizing adaptive power in neuroevolution

**DOI:** 10.1371/journal.pone.0198788

**Published:** 2018-07-18

**Authors:** Paolo Pagliuca, Nicola Milano, Stefano Nolfi

**Affiliations:** Institute of Cognitive Sciences and Technologies - National Research Council (CNR), Roma, Italia; Liverpool John Moores University, UNITED KINGDOM

## Abstract

In this paper we compare systematically the most promising neuroevolutionary methods and two new original methods on the double-pole balancing problem with respect to: the ability to discover solutions that are robust to variations of the environment, the speed with which such solutions are found, and the ability to scale-up to more complex versions of the problem. The results indicate that the two original methods introduced in this paper and the Exponential Natural Evolutionary Strategy method largely outperform the other methods with respect to all considered criteria. The results collected in different experimental conditions also reveal the importance of regulating the selective pressure and the importance of exposing evolving agents to variable environmental conditions. The data collected and the results of the comparisons are used to identify the most effective methods and the most promising research directions.

## 1. Introduction

Neuroevolution, namely the evolution of neural networks selected for the ability to perform a given function [1], constitutes a general and effective method that can be applied to a wide range of problems. It presents several advantages with respect to alternative training methods for neural networks. In particular, since it does not depend on gradient information, it can be applied to problems in which this information is unavailable, too noisy, or too costly to be obtained. By operating simply on the basis of a scalar fitness value that rates the extent to which the network solves the given problem, it can be applied to any problem. It is effective in partially observable domains since, unlike most Reinforcement Learning methods, does not rely on the Markov assumption. Finally, it can be applied to any type of neural network and can be used to adapt all the characteristics of the network including the architecture of the network, the transfer function of the neurons, and the characteristics of the system (if any) in which the network is embedded.

For these reasons, after the pioneering research carried out in the early 90s, the research in this area kept expanding over the years (for reviews see [1–3]). This has led to the development of many alternative methods that we will briefly review in the following section and to the development of several techniques that can be used to improve the efficacy of these methods (e.g. incremental evolution, see for example [4–6]).

The analysis and the data reported in the literature to date, however, do not enable to evaluate in an objective manner which are the most effective methods and which are the strengths and weaknesses of alternative methods. Progressing our understanding of these aspects is essential for successfully applying this methodology to concrete problems and for developing better methods.

In this paper we carry on a systematic comparison of the adaptive power of some of the most promising methods described in the literature including two new methods designed by us. With the term adaptive power we mean the ability to discover solutions that are robust to variations of the environment and the ability to scale-up to more complex versions of the problem. This is realized by: (i) using in a consistent manner a widely recognized benchmark problem known as the double-pole balancing problem [7], (ii) identifying and using extended versions of the problem that highlight differences in performance and in the ability to scale up to harder problems, and (iii) verifying the possibility to evolve agents able to operate effectively in varying environmental conditions.

The results obtained indicate that widely used methods such as NEAT [8] are not really competitive with the best methods identified by our analysis with respect to all criteria considered (i.e. robustness to environmental variations, speed, and capability to scale up to more complex problems).

Moreover, the analysis performed indicates that the methods proposed to date that enable to adapt also the topology of the network have a lower adaptive power than the methods that operate with fixed topology, at least in the considered problem domain.

Finally, we demonstrate how environmental variations and the addition of a moderate level of noise in the fitness function facilitate the evolution of better solutions.

## 2. Brief review of research in neuroevolution

After the first pioneering works that set up the basis of the methodology [9–13] the research in the area addressed different aspects.

A first line of research investigated the characteristics of evolutionary algorithms. In particular, [14–15] claimed the superiority of evolutionary strategy methods (that use small populations, steady state selection, and that rely primarily on mutations to generate variations) over genetic algorithms (that use large populations, generational selection, and that rely primarily on recombination to generate variations). This can be explained by considering that, in the context of neuroevolution, any phenotype (i.e. any network computing a given function) can be generated by a large number of alternative genotypes. Consequently, evolution can keep exploring the search space and improving the fitness of the population over the long-term even when genetic diversity of the population is limited [14].

A second line of research investigated the combination of evolution and learning [12–13, 16–21]. The results collected indicate that the combination of evolution and learning can be advantageous when the task/environmental conditions vary during the operation of the network [22–23]. There is not clear evidence, however, that methods combining evolution and learning outperform methods operating on the basis of evolution only when the characteristics of the adaptive problem are stable, at least when the computational cost is maintained constant. Also, further research have demonstrated how the ability to adapt to varying environmental conditions can also be obtained through other methods, namely by evolving networks with recurrent connections [24–25], and/or by evolving networks including regulatory mechanisms [26–27].

A third line of research investigated methods that enable to adapt not only the connection weights but also the topology of the neural network [8, 28–31]. A clear advantage of these methods is that they free the experimenter from the need to determine the topology of the network in advance. Whether these methods produce better or worse performance with respect to methods in which the topology of the network is fixed, however, is an open question. The results reported by researchers that carried a comparison are in fact contradictory. In particular, Stanley and Miikkulainen reported that in the case of NEAT [8] the ability to adapt the topology of the network leads to better performance with respect to a control experiment in which the topology is maintained fixed. On the other hand, Igel [32] showed how his method, which operates on the basis of a fixed topology, outperformed other methods operating with variable topology.

The NeuroEvolution of Augmenting Topology (NEAT) method proposed by Stanley and Mikkulaien [8] also introduced the following innovations: (i) a special genetic operator that allows the number of neurons to increase by minimizing the risk of performance loss, (ii) a marking system combined with a crossing over operator that allows to generate offspring by combining only the genes of the parents that are homologous, and (iii) a fitness sharing technique [33] that protects individuals with recent varied topology from the effect of selection and aims to maximize population diversity. For a more detailed description of this method see Section 3.2.

Another method that can be used to evolve the topology of the network is the Cartesian Genetic Programming of Artificial Neural Network (CGPANN) method [31, 34–35] that represents an application to neural evolution of a method widely used in Genetic Programming [36]. For more details see Section 3.4.

A fourth line of research investigated the use of cooperative evolution, which operates by evolving solution components instead of full solutions. In this manner, the overall problem of finding a solution network is broken into several smaller sub-problems. The first method of this type was the Symbiotic Adaptive NeuroEvolution (SANE) method [37] that operates by evolving a population of neurons instead of complete networks. The genotype of each neuron encodes a series of connection definitions that describe, for each connection, the neuron with which the connection is made and the weight of the connection. During each evaluation, N neurons are selected randomly and combined to form a neural network. The network is evaluated and the fitness obtained is added to the fitness of each selected neuron. The process continues until each neuron has participated in a sufficient number of networks. The neurons are then combined to form the hidden layers of neural networks that are evaluated for the capability to solve a given problem. SANE also involves the maintenance of a population of blueprints that keeps a record of the most effective neurons combinations found with the current population of neurons and uses this knowledge as the basis to form the neural networks in the next generations. The Enforced Sub-Population (ESP) method [5] consists of a variation of the previous method in which candidate solutions for evolving neurons are separated in different sub-populations, in which networks are formed by combining one candidate solution from each sub-population, and in which blueprints are not used. Finally, the Neural Evolution through Cooperatively Coevolved Synapses (CoSyNE) method [38] constitutes a variation of the ESP method that operates by evolving sub-populations of connection weights instead of sub-populations of neurons with their associated connection weights.

A fifth research line investigated the utilization of covariance matrix adaptation evolutionary strategies in which candidate solutions are sampled according to a multivariate normal distribution encoded in a matrix that is updated on the basis of the outcomes of the sampling evaluations [32, 39]. The potential strength of these methods is constituted by the possibility to adapt the sampling to the characteristics of the local portion of the search space.

Finally, a sixth research line investigated the utilization of indirect encoding methods in which the genotype does not encode directly the characteristics of the neural network, but rather the characteristics of generative rules that in turn determine the characteristic of the network and eventually of the agent in which the network is embedded. One approach consists in the utilization of genetically encoded growing rules that determine how an initial embryonic structure grows into a full-formed agent and network that is then evaluated [40–43]. An alternative approach consists in the utilization of a genetically encoded neural network that is used to generate a second network that is then evaluated [44–45].

In many of these works [8, 30–32, 38–39] the authors used the double-pole balancing problem [7] to verify the efficacy of their methods and to compare alternative methods. Consequently this problem becomes a universally recognized benchmark. Unfortunately, however, this benchmark was used only to compare the speed required by the alternative methods to find sufficiently good solutions.

In this paper, instead, we compare the most promising methods also with respect to: (i) the ability to discover solutions that are robust to environmental variations, and (ii) the ability to scale-up to complexified versions of the problem.

## 3. Method

In this section we describe the classic double-pole balancing problem and the complexified versions of the problem that we used to carry out our analysis. Then we describe the six neuroevolutionary methods that we compared, namely: NeuroEvolution of Augmenting Topologies (NEAT, see [8]), Neural Evolution through Cooperatively Coevolved Synapses (CoSyNE, see [38]), Cartesian Genetic Programming of Artificial Neural Network (CGPANN, see [34–36]), Exponential Natural Evolutionary Strategy (xNES, see [39]), and two additional methods that we developed. We selected the former four methods since they produced the best results in previous comparative analyses.

### 3.1 The double-pole balancing problem

**Fig 1:**
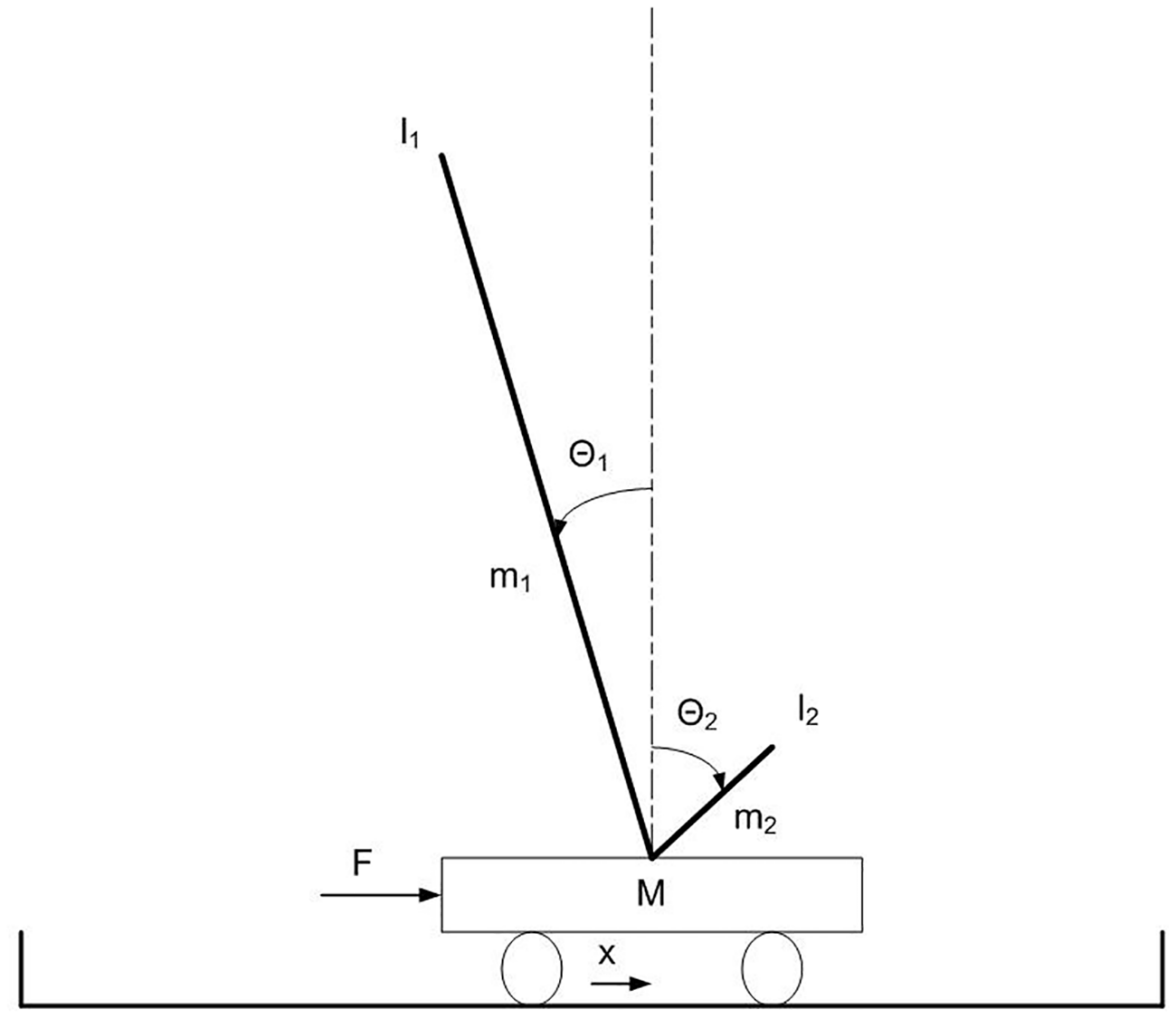
The double-pole balancing problem.

The pole balancing problem, introduced by [7], consists in controlling a mobile cart with one or two poles attached through passive hinge joints on the top of the cart for the ability to keep the poles balanced (Fig 1). This problem became a commonly recognized benchmark for the following reasons: (i) it involves fundamental aspects of agent’s control (e.g., situatedness, non-linearity, temporal credit assignment [46]), (ii) it is intuitive and easy to understand, and (iii) it requires a low computational cost.

The cart has a mass of 1Kg. The long pole and the short pole have a mass of 0.5 and 0.05 Kg and a length of 1.0 and 0.1 m, respectively. The cart can move along one dimension within a track of 4.8 m. In this paper we consider only the non-markovian version of the problem, in which the cart is provided with three sensors that encode the current position of the cart on the track (*x*), and the current angle of the two poles (θ_1_ and θ_2_). The motor controls the force applied to the cart along the x-axis. The goal is to control the force applied to the cart so to maintain the angle of the poles and the position of the cart within a viable range (details provided below).

The following equations [7] are used to compute: the effective mass of the poles (1), the acceleration of the poles (2), the acceleration of the cart (3), the effective force on each pole (4), the next angle of poles (5), the velocity of the poles (6), the position of the cart (7), and the velocity of the cart (8), respectively:

$${\hat{m}}_{i}=m_{i}(1-\frac{3}{4}{cos}^{2}\theta_{i})$$ (1)

$${\ddot{\theta }}_{i}=-\frac{3}{4l_{i}}(\ddot{x}\;cos\;\theta_{i}+g\;sin\;\theta_{i}+\frac{\mu_{pi}{\dot{\theta }}_{i}}{m_{i}l_{i}})$$ (2)

$$\ddot{x}=\frac{F+\sum_{i=0}^{N}{\hat{F}}_{i}}{M+\sum_{i=0}^{N}{\hat{m}}_{i}}$$ (3)

$${\hat{F}}_{i}=m_{i}l_{i}{\dot{\theta }}_{i}^{2}sin\theta_{i}+\;\frac{3}{4}m_{i}\;cos\;\theta_{i}(\frac{\mu_{pi}{\dot{\theta }}_{i}}{m_{i}l_{i}}+g\;sin\;{\dot{\theta }}_{i})$$ (4)

$$x[t+1]=x[t]+\;\tau \dot{x}[t]$$ (5)

$$\dot{x}[t+1]=\dot{x}[t]+\;\tau \ddot{x}[t]$$ (6)

$$\theta [t+1]=\theta [t]+\;\tau \dot{\theta }[t]$$ (7)

$$\dot{\theta }[t+1]=\dot{\theta }[t]+\;\tau \ddot{\theta }[t]$$ (8)

where *x* is the position of the cart on the track that varies in the range [-2.4, 2.4] m, $\dot{x}$ is the velocity of the cart, θ_1_ and θ_2_ are the angular positions of the poles in rad, ${\dot{\theta }}_{1}$ and ${\dot{\theta }}_{2}$ are the angular velocities of the poles, in rad/s. The dynamics of the system was simulated by using the Runge-Kutta fourth-order method and a step size of 0.01s.

The controller of the agent is constituted by a neural network with three sensory neurons and one motor neuron. The sensory neurons encode the position of the cart (*x*), and the angular position of the two poles (θ_1_ and θ_2_). The state of the *x*, θ_1_ and θ_2_ sensors are normalized in the [-0.5,0.5] m, [$-\frac{5\pi }{13.5},\;\frac{5\pi }{13.5}$] rad and [-$-\frac{5\pi }{13.5},\;\frac{5\pi }{13.5}$] rad ranges, respectively. The activation state of the motor neuron is normalized in the range [-10.0, 10.0] N and is used to set the force applied to the cart. The state of the sensors, the activation of the neural network, the force applied to the cart, and the position and velocity of the cart and of the poles are updated every 0.02 s.

To promote the evolution of solutions that are robust with respect to the initial position and velocity of the cart and of the poles, we evaluated each controller for 8 trials that varied with respect to the initial state of the system. In a first experimental condition (fixed initial states condition) we used the initial states reported in Table 1. In a second experimental condition (randomly varying initial states condition) the initial states were set randomly during each trial in the range described in Table 2.

**Table 1:** Initial states used during different trials carried out in the Fixed Initial States condition.

Trial	*x*	$\dot{x}$	θ_1_	θ_2_	${\dot{\theta }}_{1}$	${\dot{\theta }}_{2}$
1	-1.944	0	0	0	0	0
2	1.944	0	0	0	0	0
3	0	-1.215	0	0	0	0
4	0	1.215	0	0	0	0
5	0	0	-0.10472	0	0	0
6	0	0	0.10472	0	0	0
7	0	0	0	-0.135088	0	0
8	0	0	0	0.135088	0	0

**Table 2:** Range of the states used to set the initial state in the Randomly Varying Initial States condition.

	Min	max
*x*	-1.944	1.944
$\dot{x}$	-1.215	1.215
θ_1_	-0.10472	0.10472
θ_2_	-0.135088	0.135088
${\dot{\theta }}_{1}$	-0.10472	0.10472
${\dot{\theta }}_{2}$	-0.135088	0.135088

Trials terminate after 1000 steps or when the angular position of one of the two poles exceeded the range [$-\frac{\pi }{5},\;\frac{\pi }{5}$] rad or the position of the cart exceed the range [-2.4, 2.4] m. In the case of the classic double-pole problem, trials terminate after 100,000 steps.

The fitness of the agent corresponds to the fraction of time steps in which the agent maintains the cart and the poles within the allowed position and orientation ranges and is calculated on the basis of the following equations:

$$f_{i}=\frac{t}{1000}$$ (9)

$$F=\frac{\sum_{i=1}^{8}f_{i}}{8}$$ (10)

where *t* is the time step in which the cart or the pole exceeded the allowed range or 1000 in case they are maintained in the range until the end of the trial, *f*_*i*_ is the fitness of a trial, and F is the total fitness.

In the majority of the previous studies, the networks were evaluated for a single trial. We decided to use multiple trials since this forces the evolutionary process to find solutions that are robust with respect to variations of the initial state. Furthermore, some of the previous studies were carried out by using the fitness function devised by [47], usually referred with the term *damping fitness*, that includes an additional fitness component that is maximized by minimizing the value of *x*, $\dot{x}$, θ_1_, and ${\dot{\theta }}_{1}$. This additional component has been introduced to facilitate the evolution of networks capable of estimating the current velocity of the cart on the basis of the available sensor information. We did not use this additional fitness component since we did not want to reduce the complexity of the problem. Some authors claimed that this additional component prevents the discovery of brittle solutions that exploit rapid oscillations of the cart. This type of solution, however, never occurs in experiments in which the agents are evaluated for multiple trials that vary with respect to the initial state of the cart (see S1–S6 Figs). Finally, in previous studies, the generalization ability of the evolved networks has been tested by post-evaluating them for 625 trials during which the initial state of the *x*, $\dot{x}$, θ_1_ and ${\dot{\theta }}_{1\;}$ variables was varied systematically within the following values (-1.944, -1.08, 0.0, 1.08, 1.944) m, (-1.215, -0.675, 0.0, 0.675, 1.215) m/s, (0.056548, -0.031416, 0.0, 0.031416, 0.056548) rad, (-0.135088, -0.075049, 0.0, 0.075049, 0.135088) rad/s, respectively. The state of the θ_2_ and ${\dot{\theta }}_{2}$ variables was initialized to 0.0. We rather decided to post-evaluate the network on 1000 trials with initial state randomly generated in the interval described in Table 2. This allows us to verify the generalization ability of the network in a wider range of conditions.

Finally, to increase the complexity of the problem further, we also used two complexified versions of the problem. In the delayed pole-balancing problem the agent needs to determine the state of the motor on the basis of the state that the cart had 0.02s before (i.e., we take into account the fact that in hardware sensors would take time to extract information from the environment). In the long double-pole balancing problem, instead, the length and the mass of the second pole is set to 0.5m and 0.25 Kg, respectively, instead than to 0.1m and 0.05 Kg. The elongation of the short pole makes the problem significantly more challenging. Indeed, as pointed out by [7], the longer the second pole is, the higher the complexity of the problem becomes.

In the next sections we describe the neuroevolutionary methods that we tested. Some of the methods operate on fixed network topologies while others permit to evolve both the connection weights and the architecture of the neural controller. In all cases the neural network controller includes 3 sensory neurons and 1 motor neuron, as described above.

### 3.2 The NeuroEvolution of Augmenting Topologies (NEAT) method

Neuroevolution of augmenting topologies (NEAT) is a method that permits the evolution of both the weights and the topology of the neural network [8]. It is characterized by the development of progressively more complex topologies from simple networks including only sensory and motor neurons, by the utilization of innovation numbers associated to genes that permit to cross over networks with varying topologies, and by the utilization of speciation and fitness sharing [33] to preserve innovations.

The initial population is composed of a vector of *μ* genotypes of equal length. In the case of the double-pole problem each genotype includes four genes that encode four corresponding connections from the three sensory neurons and the bias to the motor neuron. Each gene contains four numbers that encode the ID number of the neurons that send and receive the connection, the weight of the connection, and the history marker constituted by a progressive innovation number.

Offspring are generated on the basis of the following genetic operators: (i) a crossing-over operator that uses the innovation numbers to identify the genes encoding the same structure in the two reproducing parents, (ii) an add-node genetic operator that replaces an existing connection genes with two new connections genes that connect the original input neuron to a new internal neuron with a weight of 1.0 and the new internal neuron to the original output neuron with a weight equal to the weight of the original connection, (iii) a mutate-weight genetic operator that perturbes the weight of each connection of the network with a given probability, and (iv) an add-connection operator that adds a connection between two pre-existing neurons.

For more details see [8]. The authors later proposed new extended methods such as HyperNEAT [45, 48] that, however, is targeted to the evolution of large neural networks and thus has not been tested on the double-pole balancing problem.

NEAT includes many parameters: crossing-over rate, add-neuron rate, add-connection rate, perturbe-weight rate, interspecies migration rate, etc. [8]. The data reported in this paper have been collected by using the author’s source code freely available and the parameters used from the authors to solve the double-pole balancing problem. The experiments were repeated by varying the size of the population in the range [20, 100, 500, 1000] and the add-neuron rate in the range [1%, 10%]. The modifications introduced in the source code to evaluate the evolving agents for 8 trials on the basis of the fitness function described in Section 3.1 are described in S1 File.

### 3.3 Neural Evolution through Cooperatively Coevolved Synapses (CoSyNE)

The neural evolution through cooperatively coevolved synapses (CoSyNE) is a method that operates by evolving solution components instead of full solutions. More precisely, instead of a population of complete neural networks, a population of network fragments is evolved [38]. Each fragment (connection weight) is evaluated as one part of a full network. The fitness of a fragment indicates how well it cooperates with other fragments of the solutions it contributes to compose.

The method operates by using **θ** sub-populations each containing *m* real numbers, where **θ** is equal to the number of connection weights and biases of the neural network and *m* is the number of alternative candidate solutions for each weight or bias. The population therefore consists of a matrix with **θ** rows and *m* columns, where each row corresponds to a sub-population. During each generation the algorithm: (i) generates *m* complete networks on the basis of the value contained in each column of the matrix, (ii) evaluates the networks, (iii) uses the top quarter networks to generate a pool of offspring that are sorted by fitness and used to replace the least fit weights of the corresponding sub-populations, and (iv) permutes the positions of the weight values in each subpopulation so to ensure that the complete network formed during the next generation are constituted by different combination of weights. For more details see [38].

The data reported in this paper have been collected by using the authors’ source code. We systematically varied the mutation rate and the number of sub-population in the following ranges [1%, 2%, 5%, 10%, 20%, 40%] and [15, 30, 60], respectively. The modifications of the source code introduced to evaluate evolving agents for 8 trials on the basis of the fitness function described in Section 3.1 are described in S2 File.

### 3.4 Cartesian Genetic Programming of Artificial Neural Network (CGPANN)

Cartesian Genetic Programming is an evolutionary method developed by Miller and Thomson [36] which has been widely used for the evolution of digital circuits and that has been recently applied also to the evolution of neural networks [31, 34–35]. It is a graph-based form of Genetic Programming that can be used to evolve the weights and the topology of neural networks.

The genotype of individuals is constituted by a list of blocks that encode the property of the internal neurons of the network. More specifically, each block encodes, with integer numbers, the ID index of the input and/or internal neurons from which the current neuron receive connections and, with real numbers, the corresponding connection weight. Moreover, the genotype includes additional blocks that encode the ID indexes of the internal neurons that are used to produce the output of the network. The length of the genotype and consequently the number of neurons and the number of incoming connections per neuron is fixed. In the initial population the integer and the real numbers contained into blocks are generated randomly within the appropriate range.

The evolutionary process is realized on the basis of (1 + *λ*) evolutionary strategy that uses a population composed of a single individual. During each generation the algorithm: generates *λ* offspring, evaluates the parent and the offspring, and selects the best individual between the parent and the offspring as the new parent.

Offspring are generated by creating a copy of the genotype of the parent and by subjecting each number contained in each block to mutations with a *MutRate* probability. Mutations are realized by replacing the real numbers, encoding the connection weights, with a new randomly generated real number on the same range or by replacing integer numbers, encoding the index of neurons projecting and receiving connections, with a new integer number generated randomly in the appropriate range. The *RecurrentConnectionsProbability* parameter is used to determine the probability with which the index of the incoming connection is replaced with a number selected in the range that includes sensory neurons or in the range that includes internal neurons.

As suggested by the authors [34–35], the number of offspring was set to 4 and the probability to generate recurrent connections was set to 20%. The number of internal neurons was set to 10. The mutation rate was varied systematically within the interval [1%, 3%, 5%, 7%, 10% and 20%]. The number of incoming connections was varied within the interval [4–12].

In one of their studies, the authors have applied this method to the double-pole balancing task [34]. In this work, however, they introduced a series of modifications that facilitate the problem. More specifically: (i) they added a new input unit that provides a function of the state of the motor neuron at time t_-1_, (ii) they used a motor neuron that encodes only two alternative torque forces (i.e. -10 N or 10 N) instead of a force varying continuously within these two values, and (iii) they used a restricted version of the algorithm that is able to synthesize feed-forward network topologies only. For these reasons we decided to use the CGPANN model described in [35] that allows the evolution of recurrent neural network and we maintained the characteristics of the problem consistent with those used in other studies.

The experiments that we performed with this method can be replicated by downloading and installing FARSA (S3 File) and the experimental plugin (S4 File).

### 3.5 The Exponential Natural Evolutionary Strategy (xNES) method

The Exponential Natural Evolutionary Strategy (xNES) was proposed by Wierstra, Schaul, Peters, and Schmidhuber [39] as an extension of Natural Evolutionary Strategy originally proposed by [49–50], see also [32]. xNES operates on the basis of a single candidate solution that consists of a vector of **θ** real numbers and on the basis of a **θ** x **θ** co-variance matrix that encodes the correlation between the variation of the fitness and the variation of the **θ** parameters. The genes of the candidate solutions are initialized randomly. The co-variance matrix is initialized with all zero values.

During each generation the algorithm: (i) generates the variation vectors that are constituted by *λ* vectors of **θ** real numbers generated randomly with a Gaussian distribution, (ii) generates *λ* varied candidate solutions that are obtained by adding to the candidate solution the variation vectors multiplied by the co-variance matrix, (iii) evaluates the varied candidate solutions, (iv) estimates the local gradient of the candidate solutions on the basis of the fitness of the varied candidate solutions and uses the gradient to update the covariance matrix, and (v) modifies the current candidate solution in the direction of the estimated local gradient for a given length or learning rate. The number of candidate solutions is a constant that is proportional to the number of parameters to be adapted and is set to 19 in the case of our experiments that involve 151 parameters. For more details see [39].

The only parameter of the method is the learning rate that, according to the authors, should be set to 1.0 [39]. By systematically varying the learning rate with the following values [0.1, 0.2, 0.25, 0.5, 1.0], however, we observed that the best results are obtained with values included in the following range [0.1, 0.5], see below.

The experiments that we performed with this method can be replicated by downloading and installing FARSA (S3 File) and the experimental plugin (S5 File).

### 3.6 The Stochastic Steady State (SSS) method

The Stochastic Steady State (SSS) is a (*μ* + *μ*) evolutionary strategy [49] that operates on the basis of populations formed by *μ* parents. During each generation each parent generates one offspring, the parent and the offspring are evaluated, and the best *μ* individuals are selected as new parents (see the pseudo-code below). It is a method developed by us that belongs to the class of methods proposed by [14–15]. The novelty with respect to previous related methods consists in the introduction of the Stochasticity parameter that permits to regulate the selective pressure. This is obtained by adding to the fitness of individuals a value randomly selected in the range [-Stochasticity*MaxFitness, Stochasticity*MaxFitness] with a uniform distribution. When this parameter is set to 0.0 only the best *μ* individuals are allowed to reproduce. The higher the value of the parameter is, the higher the probability that other individuals reproduce is. For a discussion of evolutionary optimization in uncertain environments and in the presence of a noisy fitness function see [51].

**SSS Method**:

1: NEvaluations <- 0

  // the genotype of the parents of the first generation in initialized randomly

2: **for** *p* *<-* *0* **to** *NParents* **do**

3:   **for** *g <- 0* **to** *NGenes* **do**

*4*:     genome[p][g] <- rand(-8.0, 8.0)

5:   Fitness[p] <- evaluate (*p)*

6:   NEvaluations <- NEvaluations + NTrials

  // evolution is continued until a maximum number of evaluation trials is performed

7: **while**(NEvaluations < MaxEvaluations) **do**

8:   **for** *p <- 0* **to** *NParents* **do**

     // in the randomly varying experimental condition parents are re-evaluated

9:    **if** (RandomlyVaryingInitialCondition) **then**

10:     Fitness[p] <- evaluate (*p)*

11:     NEvaluations <- NEvaluations + NTrials

12:    genome[p+NParents] <- genome[p] // create a copy of parents’ genome

13:    mutate-genome(p+NParents) // mutate the genotype of the offspring

14:    Fitness[p+Nparents] <- evaluate[p+NParents]

15:    NEvaluations <- NEvaluations + NTrials

   // noise ensures that parents are replaced by less fit offspring with a low probability

16:      NoiseFitness[p] <- Fitness[p] * (1.0 + rand(-Stochasticity*MaxFit, Stochasticity*MaxFit))

17:    NoiseFitness[p+NParents] <-

         Fitness[p+NParents] * (1.0 + rand(-Stochasticity*MaxFit, Stochasticity*MaxFit))

     // the best among parents and offspring become the new parents

18:  **rank** genome[NParents*2] **for** NoiseFitness[NParents*2]

In the experiment reported in this paper the connection weights are evolved and the topology of the network is kept fixed. The initial population is encoded in a matrix of *μ* x **θ** real numbers that are initialized randomly with a uniform distribution in the range [-8.0, 8.0], where *μ* corresponds to the number of parent and **θ** corresponds to the total number of weights and biases. Offspring are generated by creating a copy of the genotype of the parent and by subjecting each gene to mutation with a *MutRate* probability. Mutations are realized by replacing or perturbing a gene value with equal probability. Replacements are realized by substituting the gene with a new real number randomly generated within the range [-8.0, 8.0] with a uniform distribution. Perturbations are realized by adding to the current value of a gene a real number randomly selected with a Gaussian distribution with a standard deviation of 1.0 and a mean of 0.0. Values outside the [-8.0, 8.0] range after perturbations are truncated in the range.

This method requires setting two parameters: *MutRate* and *Stochasticity*. To identify the optimal values of the parameters we systematically varied *MutRate* in the range [1%, 3%, 5%, 7%, 10%, 15% and 20%] and *Stochasticity* in the range [0%, 10%, 20%, 30%, 40%, 50%, 70%, and 100%]. Population size was set to 20. To enhance the ability of the method to refine the evolved candidate solutions, we reduced the mutation rate to 1% and we set the Stochasticity to 0% during the last 1/20 period of the evolutionary process.

The experiments that we performed with this method can be replicated by downloading and installing FARSA [52–53] (S3 File) and the experimental plugin (S6 File).

### 3.7 Parallel Stochastic Hill Climber (PSHC and PSHC*)

The Parallel Stochastic Hill Climber (PSHC) is a new method that we developed that consists of a combination of a (*μ* + *μ*) and a (1 + 1) evolutionary strategy. The novelty of this method consists in the utilization of a stochastic hill climber method operating on single individuals that maximizes exploration at the risk of retaining maladaptive variations combined with an evolutionary strategy operating on a population of stochastically hill climbing individuals which ensures the retention of acquired adaptive traits.

The initial population is composed by a matrix of *μ* x **θ** real numbers that are initialized randomly with a uniform distribution in the range [-8.0, 8.0], where *μ* corresponds to the number of parent and **θ** corresponds to the total number of weights and biases. Varied individuals are generated by creating a copy of the genotype of the original individual and by subjecting each gene to mutation with a *MutRate* probability. Mutations are realized by replacing or perturbing a real number with equal probability. Replacements are realized by substituting the gene with a new real number randomly generated within the range [-8.0, 8.0] with a uniform distribution. Perturbations are realized by adding to the gene a real number randomly selected with a Gaussian distribution with a standard deviation of 1.0 and a mean of 0.0. Values outside the [-8.0, 8.0] range after perturbations are truncated in the range.

Each generation involves an adaptation phase, during which each individual is adapted for a certain number of *Variations* through a (1 + 1) evolutionary strategy and a selection phase in which part of the adapted individuals are used to compose the new population.

During the adaptation phase, for each individual of the population, a sequence of N varied individuals are created and evaluated during N corresponding variation steps. The first varied individual is generated by creating a mutated copy of the original individual. The other varied individuals are generated by creating a mutated copy of the last varied individual that matched or outperformed the original individual (or by creating a mutated copy of the original individual when none of the previous varied individuals matched or outperformed the original individual). During this phase, the fitness of the individuals is perturbed through the addition of a value randomly selected in the range [-Stochasticity*MaxFitness, Stochasticity*MaxFitness] with a uniform distribution.

During the selection phase, for each individual of the population, the best of its variations is selected for the new population. Moreover the best-selected variation of all individuals is used to replace the worst selected variation with an *Interbreeding* probability. Contrary to the adaptation phase, the selection phase is realized on the basis of the real fitness (i.e. the fitness without the addition of noise). The pseudo-code of the method is included below.

**PSHC Method**:

1: NEvaluations <- 0

   // the genotype of the parents of the first generation is initialized randomly

2: **for** *p <- 0* **to***NParents* **do**

3:   **for** *g <- 0* **to** *NGenes* **do**

4:     Genome[p][g] <- rand(-8.0, 8.0)

5:    Fitness[p] <- evaluate (*p)*

6:     NEvaluations <- NEvaluations + NTrials

   // evolution is continued until a maximum number of evaluation trials is performed

7: **while** (NEvaluations < MaxEvaluations) **do**

8:   **for** *p <-0* **to***NParents* **do**

             // each individual is adapted through a (1 + 1) ES

9:           VariedGenome[0] <- Genome[p]

10:          VFitness[0] <- evaluate (VariedGenome[0])

11:          NEvaluations <- NEvaluations + NTrials

12:          **for** *v <- 0* **to** *NVariations* **do**

13:            VariedGenome[v+1] <- VariedGenome[v]

14:            mutate-genome(VariedGenome[v+1]);

15:            VFitness[v+1] <- evaluate(VariedGenome[v+1])

16:            NEvaluations <- NEvaluations + NTrials

                 // maladaptive variations are discarded

                 // noise ensure that occasionally maladaptive variations are retained

17:            **if**(Vfitness[v+1]*(1.0 + rand(-Stochasticity*MaxFit, Stochasticity*MaxFit)) <

              VFitness[v]*(1.0 + rand(-Stochasticity*MaxFit, Stochasticity*MaxFit)) **then**

18:              VariedGenome[v+1] <- VariedGenome[v]

             // the best varied genotype is selected

20:               **rank** VariedGenotype[NVariations] **for** fitness[NVariations]

            // the original genotype is replaced with its best variation

21:         Genome[p] <- VariedGenotype[p]

22:         Fitness[p] <- VFitness[p]

           // the worse individual of the population is occasionally replaced with the best individual

23:        **rank** Genome[NParents] **for** Fitness[NParents]

24:         **if** (rand(0.0,1.0) > Interbreeding) **and** (Fitness[0] > = Fitness[Nparents-1]) **then**

25:          Genome[NParents-1] <- Genome[0]

The PSHC* method is a variation of the PSHC method that allows to evolve also the topology of the network. The difference only concerns the mutation operator. In the case of the PSHC* method mutations are realized by setting connection weights to a null value (i.e., by eliminating weights) with a *EliminateConnection* probability and by replacing or perturbing the connection weight as usual with the remaining probability. Mutations of connections with null weight value cause the creation of new connections or the re-establishment of previously eliminated connections.

The PSHC method requires setting three parameters: *MutRate*, *Stochasticity* and *Interbreeding*. To identify the optimal values of the parameters we systematically varied *MutRate* in the range [1%, 3%, 5%, 7%, and 10%] and *Stochasticity* in the range [0%, 10%, 20%, 30%, 40%, 50%, 70%, and 100%]. The *Interbreeding* parameter was set to 10%.

To enhance the ability of the method to refine the evolved candidate solutions, we reduced the mutation rate to 1% and we set the Stochasticity to 0% during the last 1/20 period of the evolutionary process. The population size was set to 20.

The experiments that we performed with this method can be replicated by downloading and installing FARSA [52–53] (S3 File) and the experimental plugin (S7 File).

## 4. Results

Table 3 displays the results obtained on the classic non-markovian version of the double-pole balancing problem used in previous comparisons. In these experiments agents were evaluated for a single trial and the initial state of the cart and of the pole was set to [*x* = 0, $\dot{x}=0$, θ_1_ = 4°, θ_2_ = 0, ${\dot{\;\theta }}_{1}=0\;$, ${\dot{\theta }}_{2}=0$]. The table displays the results obtained with the best parameters, i.e. the parameters that allow minimizing the number of evaluations required to solve the problem and to find a solution in all replications (see the caption of Table 3).

**Table 3:** Evaluations required to solve the problem averaged over 50 replications of the experiments and percentage of replications that successfully solved the problem (i.e. that found a network capable of balancing the pole until the end of the trial).

Classic Double-Pole	Evaluations
xNES	395
PSHC	563
CoSyNe	1257
SSS	1557
CGPANN	6885
NEAT	7743

In the methods with fixed topologies, we used full recurrent architectures. The data reported in the table are those obtained with the best combination of parameters: xNES (fixed positions: LearningRate 0.5, NumHiddens 0), PSHC (MutRate 50%, Stochasticity 10%, Interbreeding 10%, NumHiddens 1), CoSyNE (MutRate 90%, SubPopulations 5, NumHiddens 1), SSS (fixed positions: MutRate 50%, Stochasticity 20%, NunHiddens 1), CGPANN (MutRate 3%; NumIncomingConnections 8, NumHiddens 2), and NEAT (popSize 100, NumHiddens 1.8, see Stanley and Miikkulainen [2002] for the other parameters). In the case of NEAT, the number of hidden neurons indicates the average number of internal neurons possessed by first solution obtained in each replication. The network of the fastest replication included 1 hidden neuron.

As can be seen, all methods manage to solve the problem in all replications. The results obtained with NEAT, CoSyNE and xNES are in line with those published in [38–39]. The CGPANN method was tested previously only on a simplified version of the problem (see Section 3.5). The performance statistically differs in all cases (Mann-Whitney U test, p-value < 0.05 with Bonferroni correction) with the exception of the performance of SSS versus CoSyNE (Mann-Whitney U test with Bonferroni correction, p-value > 0.05 with Bonferroni correction) and of NEAT versus CGPANN (Mann-Whitney U test, p-value > 0.05 with Bonferroni correction) that do not differ among them. Consequently, xNES results the fastest method followed by PSHC, followed by CoSyNE and SSS, followed by CGPANN and NEAT.

We now report the results obtained in the three extended versions of the double pole balancing problem (i.e. the standard, the delayed, and the long double-pole balancing problems). For each experiment we report the results obtained in the Fixed and Randomly Varying Initial States conditions. In the case of the long double-pole balancing problem we tested only the methods that produced good performance on the first two problems.

In the case of the methods that operate with fixed topology, the agents are provided with a three-layer neural network with 10 internal neurons and recurrent connections. In all cases evolution is terminated after 16 million of evaluations (i.e. after a total of 16 millions trials were performed).

**Fig 2:**
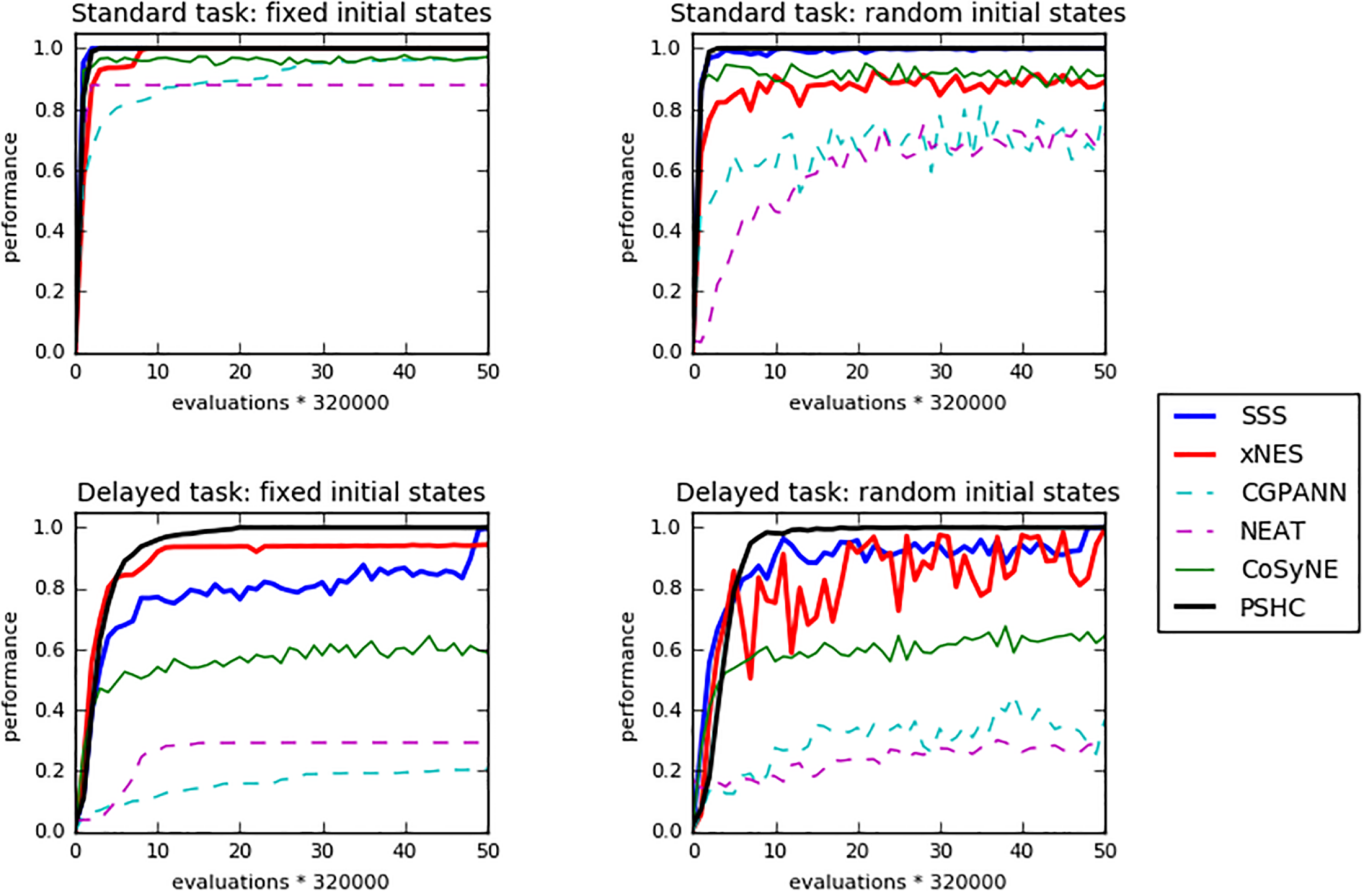
Performance of the best agents evolved through different methods during the evolutionary process. The top and bottom picture display that data of the standard and delayed double-pole problems, respectively. The left and right pictures display the data of the experiment carried out in the Fixed and Randomly varying initial conditions, respectively. Each curve displays the average result of the best 30 networks evolved in the 30 corresponding replications of each experiment. Data refer to the best individual of the population.

**Fig 3:**
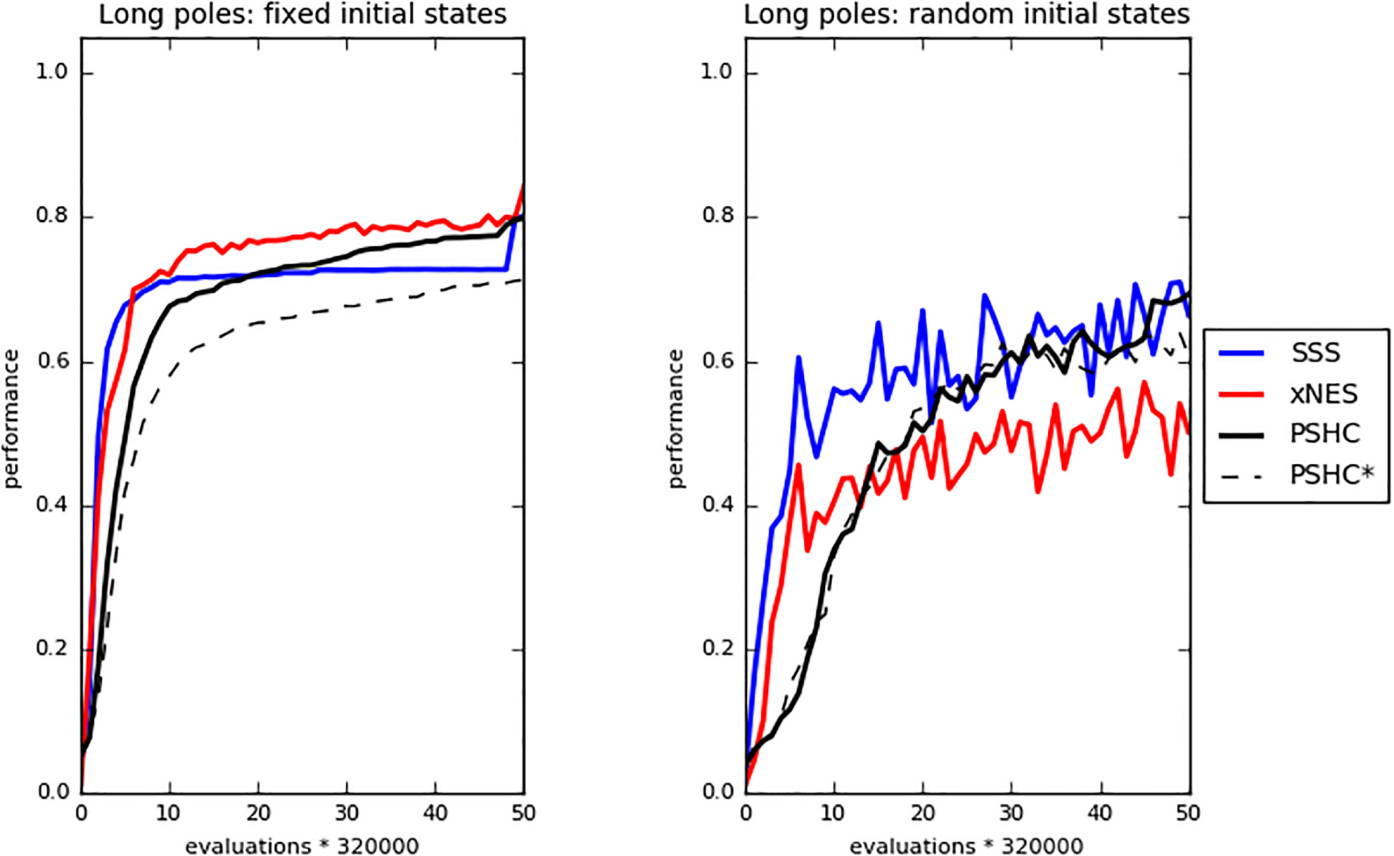
Long double-pole problem. Performance of the best agents during the course of the evolutionary process. The left and right pictures display the data of the experiment carried out in the Fixed and Randomly varying initial conditions, respectively. Each curve displays the average result of the best 30 networks evolved in the 30 corresponding replications of each experiment. Data refer to the best individual of the population.

The performance and generalization abilities of the evolved agents in the case of the standard and delayed versions of the problem are reported in Tables 4 and 5. The Fixed Initial States condition refers to the experiments in which the state of the cart at the beginning of each trial is set in a deterministic manner (see Table 1). The Randomly Varying Initial States condition, instead, refers to the experiments in which the initial state of the cart is set randomly within the range indicated in Table 2. Performance indicates the fitness obtained by the best-evolved agents evaluated from the same initial states that they experienced during evolution. Generalization indicates the fitness obtained by the best evolved agents post-evaluated for 1000 trials during which the initial state of the cart was set randomly within the ranges indicated in Table 2. The way in which performance varies during the course of the evolutionary process is reported in Figs 2 and 3. In the case of NEAT, the neural network of evolved agents include 6.6 and 6.4 internal neurons, on the average in the case of the standard double-pole (fixed and randomly varying initial states) and 9.5 and 8.9 neurons, on the average in the case of the delayed double-pole.

**Table 4:** Performance and generalization ability of neural network controllers evolved with different methods on the double-pole balancing problem.

Standard Double-Pole	Fixed Initial States		Randomly Varying Initial States	
	Performance	Generalization	Performance	Generalization
NEAT	0.879	0.397 [322]	0.696	0.710 [656]
CoSyNE	0.971	0.701 [609]	0.911	0.699 [594]
CGPANN	0.967	0.693 [622]	0.824	0.613 [506]
xNES	**1.0**	0.717 [696]	0.889	0.897 [884]
SSS	**1.0**	0.821 [791]	**1.0**	0.906 [893]
PSHC	**1.0**	0.785 [746]	**1.0**	0.807 [788]

Each number indicates the average performance obtained during 30 replications of the experiment. Generalization refers to the average performance obtained by post-evaluating the evolved networks on 1000 trials during which the initial states of the cart have been set randomly. The numbers in square brackets indicate the number of trials in which the agents manage to maintain the poles balanced for the entire duration of the trial during the post-evaluation test. The data reported in the table are those obtained with the best combination of parameters: NEAT (fixed positions: Population 1000; varying positions: Population 1000, for the other parameters see Stanley and Miikkulainen, 2002), CoSyNE (fixed positions: MutRate 5%, SubPopulation 60; varying positions: MutRate 10%, SubPopulation 60), CGPANN (fixed positions: MutRate 20%, Incoming Connections 8); varying positions: MutRate 1%, Incoming Connections 4), xNES (fixed positions: LearningRate 0.2; varying positions: LearningRate 0.1), SSS (fixed positions: MutRate 7%, Stochasticity 10%; varying positions: MutRate 3%, Stochasticity 10%), PSHC (fixed positions: MutRate 7%, Stochasticity 50%, Interbreeding 10%; varying positions: MutRate 7%, Stochasticity 0%, Interbreeding 10%).

**Table 5:** Performance and generalization ability of neural network controllers evolved with different methods on the delayed double-pole balancing problem.

Delayed Double-Pole	Fixed Initial States		Randomly Varying Initial States	
	Performance	Generalization	Performance	Generalization
NEAT	0.292	0.038 [0]	0.300	0.037 [0]
CoSyNE	0.589	0.453 [380]	0.642	0.434 [360]
CGPANN	0.206	0.077 [40]	0.367	0.284 [171]
xNES	0.943	0.692 [679]	0.992	0.911 [903]
SSS	0.996	0.756 [731]	**1.0**	0.873 [854]
PSHC	**1.0**	0.685 [635]	**1.0**	0.755 [722]

Each number indicates the average performance obtained during 30 replications of the experiment. Generalization refers to the average performance obtained by post-evaluating the evolved networks on 1000 trials during which the initial states of the cart have been set randomly. The numbers in square brackets indicate the number of trials in which the agents manage to maintain the poles balanced for the entire duration of the trial during the post-evaluation test. The best performance is indicated in bold. The data reported in the table are those obtained with the best combination of parameters: NEAT (fixed positions: Population 1000; varying positions, Population 1000, for the other parameters see Stanley and Miikkulainen, 2002), CoSyNE (fixed positions: MutRate 10%, Subpopulations 60; varying positions: MutRate 5%, Subpopulations 60), CGPANN (fixed positions: MutRate 20%, Incoming Connections 8; varying positions: MutRate 1%, Incoming Connections 5), xNES (fixed positions: LearningRate 0.2; varying positions: LearningRate 0.1), SSS (fixed positions: MutRate 3%, Stochasticity 100%; varying positions: MutRate 7%, Stochasticity 70%), PSHC (fixed positions: MutRate 7%, Stochasticity 50%; Interbreeding10%, varying positions: MutRate 3%, Stochasticity 0%, Interbreeding 10%).

As can be seen the SSS, xNES and PSHC methods outperform the NEAT, CGPANN and CoSyNE methods in the case of the standard and delayed double pole problems (Tables 4 and 5). The performance of the former three methods are significantly better than the performance of the latter three methods (Mann-Whitney U test, p-value < 0.05 with Bonferroni correction in all cases) with the exception of the performance of xNES and CoSyNE methods in the random initial states condition which do not differ statistically among themselves (Mann-Whitney U test, p-value > 0.05 with Bonferroni correction). Given the clear superiority of the former methods we did not test the latter methods on the long double-pole balancing problem.

The agents evolved with the SSS and PSHC methods also generalize better than the agents evolved with the NEAT, CGPANN and CoSyNE methods in all cases (Tables 4 and 5, Mann-Whitney U test, p-value < 0.05 with Bonferroni correction). The xNES method generalizes better than the NEAT, CGPANN and CoSyNE methods in all cases with the exception of the fixed initial states condition (Mann-Whitney U test, p-value > 0.05 with Bonferroni correction).

To analyze the speed with which the different methods find a close-to-optimal solution we compared the number of evaluations required to reach a fitness equal or greater than 0.9 for methods that reached this threshold in at least 15 out of 30 replications (see Table 6 and Fig 2). In the case of the Standard Double-Pole problem the SSS method is significantly faster than xNES and CGPANN methods (Median test, p-value < 0.05 with Bonferroni correction) and does not differ from the NEAT, CoSyNE and PSHC methods (Median test, p-value > 0.05 with Bonferroni correction). NEAT is significantly faster than xNES, CGPANN and PSHC methods (Median test, p-value < 0.05 with Bonferroni correction) and does not statistically different from CoSyNE (Median test, p-value > 0.05 with Bonferroni correction). The CGPANN, NEAT, CoSyNE and PSHC methods do not differ among themselves (Median test > 0.05 with Bonferroni correction in all cases). In the case of the Delayed Double-Pole problem, only the agents evolved with the xNES, PSHC and SSS method reach a fitness of 0.9 in 15 out of 30 replications. The speed with which they reach sufficiently good performance does not statistically differ (Median test, p-value > 0.05 with Bonferroni correction in all cases).

**Table 6:** Average number of evaluations required to reach a fitness equal or greater than 0.9 for the first time.

Fixed Initial States condition	Standard Double-Pole	Delayed Double-Pole
NEAT	234,666.6	*
CoSyNE	256,000.0	*
CGPANN	2,389,333.3	*
xNES	490,666.6	1,450,666.6
SSS	192,000.0	3,989,333.3
PSHC	405,333.3	1,902,933.3

Asterisks indicate the conditions that failed to reach this threshold in more than 15 out of 30 replications.

An additional advantage of the SSS, xNES and PSHC methods is constituted by the fact that they require to set few parameters and tend to produce good solutions for a wide range of parameters’ values, especially in the Randomly Varying Initial State conditions. The SSS method, for example, requires setting only two parameters (the mutation rate and the stochasticity range) and displays rather high performance for most combinations of parameters value (see Table 7). Data for the other methods and for the other experimental conditions are available in the supporting information (see S1–S5 Tables).

**Table 7:** Performance and generalization ability of neural network controllers evolved with the SSS method on the standard and delayed double-pole balancing problem in the Varying Initial States experimental condition in experiment carried out with different combination of parameters.

**Double-Pole**	**MutRate1%**	**MutRate3%**	**MutRate5%**	**MutRate7%**	**MutRate10%**
**Stochasticity 0%**	1.0 [0.901]	1.0 [0.899]	1.0 [0.894]	1.0 [0.890]	1.0 [0.888]
**Stochasticity 10%**	1.0 [0.897]	1.0 [0.906]	1.0 [0.897]	1.0 [0.894]	1.0 [0.888]
**Stochasticity 20%**	0.996 [0.896]	1.0 [0.896]	1.0 [0.895]	0.998 [0.881]	1.0 [0.880]
**Stochasticity 30%**	1.0 [0.894]	1.0 [0.895]	1.0 [0.881]	1.0 [0.883]	1.0 [0.881]
**Stochasticity 40%**	0.989 [0.870]	1.0 [0.893]	1.0 [0.875]	1.0 [0.874]	1.0 [0.875]
**Stochasticity 50%**	1.0 [0.887]	0.998 [0.880]	0.999 [0.870]	1.0 [0.882]	1.0 [0.872]
**Delayed Double-pole**	**MutRate1%**	**MutRate3%**	**MutRate5%**	**MutRate7%**	**MutRate10%**
**Stochasticity 0%**	0.917 [0.744]	0.956 [0.806]	0.957 [0.818]	0.962 [0.809]	0.972 [0.805]
**Stochasticity 10%**	0.894 [0.743]	0.909 [0.748]	0.977 [0.820]	0.960 [0.836]	0.988 [0.847]
**Stochasticity 20%**	0.952 [0.780]	0.993 [0.829]	0.994 [0.844]	0.969 [0.818]	0.971 [0.827]
**Stochasticity 30%**	0.965 [0.812]	0.983 [0.839]	0.969 [0.831]	0.963 [0.826]	0.966 [0.822]
**Stochasticity 40%**	0.942 [0.789]	0.989 [0.852]	0.983 [0.852]	1.0 [0.862]	0.994 [0.863]
**Stochasticity 50%**	0.982 [0.835]	0.986 [0.836]	0.993 [0.856]	0.993 [0.856]	0.980 [0.823]
**Stochasticity 70%**	1.0 [0.847]	1.0 [0.862]	0.994 [0.862]	1.0 [0.873]	1.0 [0.869]
**Stochasticity 100%**	0.988 [0.834]	0.996 [0.845]	1.0 [0.867]	0.818 [0.700]	0.350 [0.250]

Each number indicates the average performance obtained during 30 replications of the experiment. Generalization refers to the average performance obtained by post-evaluating the evolved networks on 1000 trials during which the initial states of the cart have been set randomly. The numbers in square brackets indicate the number of trials in which the agents manage to maintain the poles balanced for the entire duration of the trial during the post-evaluation test.

The differences in performance and generalization among the SSS, xNES, and PSHC methods are less marked. In the case of the long double-pole balancing problem (Table 8, Fig 3), that turned out to be much harder than the other two problems, the best performance in the Fixed Initial States condition are achieved by the xNES method (Mann-Whitney U test, p-value < 0.05 with Bonferroni correction). The performance of the SSS and the PSHC methods does not vary statistically among themselves (Mann-Whitney U test, p-value > 0.05 with Bonferroni correction). In the case of the Randomly Varying Initial States condition the best performance are achieved by the SSS and PSHC methods that differ statistically from the xNES method (Mann-Whitney U test, p-value < 0.05 with Bonferroni correction) and do not differ among themselves (Mann-Whitney U test, p-value > 0.05 with Bonferroni coorrection). The xNES method generalize significantly better than the SSS and PSHC method in the case of the Randomly Varying Initial States condition (Mann-Whitney U test, p-value < 0.05 with Bonferroni correction). In the case of the Fixed Initial States condition, instead, the generalization performance of the xNES method do not statistically differ from those of the SSS method (Mann-Whitney U test, p-value > 0.05 with Bonferroni correction).

**Table 8:** Performance and generalization ability of neural network controllers evolved with different methods on the long double-pole balancing problem.

Long double-pole	Fixed Initial States		Randomly Varying Initial States	
	Performance	Generalization	Performance	Generalization
xNES	**0.842**	0.220 [190]	0.502	0.520 [493]
SSS	0.802	0.247 [197]	0.664	0.431 [394]
PSHC	0.799	0.209 [142]	**0.693**	0.312 [245]
PSHC*	0.715	0.238[179]	0.602	0.335 [261]

Each number indicates the average performance obtained during 30 replications of the experiment. Generalization refers to the average performance obtained by post-evaluating the evolved networks on 1000 trials during which the initial states of the cart has been set randomly. The best performances are indicated in bold. The numbers in square brackets indicate the number of trials in which the agents manage to maintain the poles balanced for the entire duration of the trial during the post-evaluation test. The data reported in the table are those obtained with the best combination of parameters: xNES (fixed positions: LearningRate 0.5; varying positions: LearningRate 0.1), SSS (fixed positions: MutRate 20%, Stochasticity 30%; varying positions: MutRate 3%, Stochasticity 0%), PSHC (fixed positions: MutRate 7%, Stochasticity 50%, Interbreeding10%; varying positions: MutRate 3%, Stochasticity 0%, Interbreeding 10%), PHSC* (fixed positions: MutRate 7%, EliminateConnection 40%, Stochasticity 70%, Interbreeding 10%; varying positions: MutRate 1%, EliminateConnection 15%, Stochasticity 50%, Interbreeding 10%).

The results of the PSHC* reported in Table 8 and Fig 3 refer to the experiments carried out with the variation of the Parallel Stochastic Hill Climber method that permits to adapt also the topology of the neural network. In consideration of the fact that the networks produced by the PSHC* method are not fully connected, we set the number of internal neurons to 15 instead of 10, as in the other experiments. In the case of the Randomly Varying Initial States condition the performance and generalization capabilities obtained with this method do not differ statistically from those obtained with the standard PSHC method that operates with a fixed network topology (Mann-Whitney U test, p-value > 0.05). In the case of the Fixed Initial States condition, instead, the PSHC* method produces worse performance (Mann-Whitney U test, p-value < 0.05) and better generalization (Mann-Whitney U test, p-value > 0.05) than the standard PSHC method. The percentage of connections present in the evolved networks matches very closely the value of the *EliminateConnection* parameter. In other words, the average rate of connectivity of evolved networks is determined by setting the parameter while the specific connections that are present or absent are determined by the evolutionary process. By systematically varying the *EliminateConnection* parameter we observed that the best results are obtained by setting this parameter to 40% and 15% in the case of the experiments carried out in the Fixed and Randomly Varying Initial States experimental conditions, respectively.

We should now discuss the relationship between the variability of the environmental conditions in which the agents are evaluated during the course of the evolutionary process and the performance and generalization capabilities of the evolved agents.

An advantage of evaluating the agents in randomly varying environmental conditions is that it favors the selection of solutions that are robust with respect to these variations, i.e. solutions that generalize. The fact that the environmental conditions vary, on the other hand, implies that the fitness measure of individuals can be over or under-estimated. The fitness is over-estimated when the individual happens to be evaluated in easier than average environmental conditions and under-estimated when the individual happens to be evaluated in more complex than average conditions. The effect of this over or under-estimation is analogous to the effect of adding noise to the fitness. The addition of noise to the fitness (within limits) does not reduce the efficacy of evolution but rather promotes the evolution of better solutions (see Table 9). Consequently, the utilization of randomly varying conditions for the evaluation of the evolving agents is expected to produce better results than the utilization of fixed environmental condition.

**Table 9:** Performance and generalization ability of the agents evolved with the PSHC and the SSS method in the Fixed Initial States condition in the experiments carried out with or without stochasticity.

**Standard double-pole**	**Stochasticity = 0.0**	**Stochasticity>0**
SSS	1.0	1.0
PSHC	1.0	1.0
**Delayed double-pole**	**Stochasticity = 0.0**	**Stochasticity>0**
SSS	0.867	0.996
PSHC	0.823	1.0
**Long double-pole**	**Stochasticity = 0.0**	**Stochasticity>0**
SSS	0.760	0.802
PSHC	0.479	0.799

The numbers refer to the data obtained with the best combination of parameters, i.e. with the best mutation rate in the case of the data reported in the second column, and with the best mutation rate and stochasticity level in the case of the data reported in the third column.

This hypothesis is confirmed by the comparison of the results obtained in the Fixed and in the Randomly Varying Experimental condition (see Table 10). As expected, in fact, the agents evolved in the Randomly Varying Initial States condition generalize better than the agents evolved in the Fixed State Initial States condition in all cases. The performance in the two conditions are not comparable since the agents evolved in fixed environmental conditions tend to select solutions that are particularly effective for those conditions while agents evolved in randomly varying initial conditions cannot produce this type of over-fitting.

**Table 10:** Statistical difference between the generalization performance obtained in the Fixed and Randomly Varying Initial States conditions for the experiment carried with the xNES, SSS, and PSHC, methods.

	Fixed Initial States	Randomly Varying Initial States	Mann-Whitney U test, p-value
**Standard double-pole**			
xNES	0.717 [696]	0.897 [884]	< 0.05
SSS	0.821 [791]	0.906 [893]	< 0.05
PSHC	0.785 [746]	0.807 [788]	< 0.05
**Delayed double-pole**			
xNES	0.692 [679]	0.911 [904]	< 0.05
SSS	0.756 [731]	0.873 [854]	< 0.05
PSHC	0.685 [635]	0.755 [722]	< 0.05
**Long double-pole**			
xNES	0.220 [190]	0.520 [493]	< 0.05
SSS	0.247 [197]	0.431 [394]	< 0.05
PSHC	0.209 [142]	0.312 [245]	< 0.05

Data collected on the experiments carried with the best parameters (see Tables 4, 5 and 8). Each number indicates the average performance obtained during 30 replications of the experiment. Generalization refers to the average performance obtained by post-evaluating the evolved networks on 1000 trials during which the initial states of the cart have been set randomly. The numbers in square brackets indicate the number of trials in which the agents manage to maintain the poles balanced for the entire duration of the trial during the post-evaluation test.

The other aspect that strongly affects the performance of the evolving agents is the number of the trials used, i.e. the number of different conditions in which the agents are evaluated (see Table 11). The comparison of the results obtained by varying systematically the number of trials indicates that the best performance and generalization abilities are achieved by using a relatively small number of trials (i.e. 4–8 trials).

**Table 11:** Performance and generalization capabilities of neural network controllers evolved with the SSS method in the Randomly Varying Initial States condition by using a variable number of trials.

N. Trials	Double-Pole	Delayed Double-Pole	Long Double-Pole
1	1.0 [0.826]	1.0 [0.818]	0.180 [0.075]
2	1.0 [0.863]	1.0 [0.870]	0.458 [0.182]
4	1.0 [0.882]	1.0 [0.865]	**0.779** [0.384]
**8**	**1.0** [0.906]	**1.0**[0.873]	0.664 [0.431]
16	0.990[0.907]	0.969 [0.867]	0.683 [0.421]
32	0.968 [0.905]	0.903 [0.821]	0.493 [0.353]
64	0.917 [0.885]	0.855 [0.810]	0.325 [0.261]

Data collected by using the best parameters. The numbers in square brackets indicate the average performance obtained by post-evaluating the corresponding 30 best evolved agents for 1000 trials during which the initial state of the cart have been set randomly within the range shown in Table 2. Each number indicates the average performance obtained during 30 replications of the corresponding experiment.

This can be explained by considering that increasing the number of trials has both costs and benefits. The computational cost increases linearly with the number of trials. Consequently when the computational cost is maintained constant, as in the case of our experiments, the utilization of a larger number of trials leads to a reduction of the number of generations that impact negatively on performance. The utilization of additional trials, on the other hand, facilitates the selection of robust solutions and allows reducing the level of noise in the fitness estimation (which constitutes an advantage when the fitness is too noisy as a consequence of the fact that the fitness of the agent is evaluated on few trials).

## 5. Discussion

Neuroevolution represents a convenient technique that can be potentially applied to any type of problem. Its efficacy, however, depends on the possibility to improve candidate solutions for prolonged periods of time without entering in stagnation phases caused by the preliminary convergence into sub-optimal solutions or by the inability to keep generating and/or retaining better solutions.

The interest in this technique has led to the development of several alternative methods that we briefly reviewed. In this paper, we analyzed the adaptive power of some of the most promising methods described in the literature, including two new methods developed by us, in the context of the double-pole balancing problem. By adaptive power, we mean the capacity of a method to discover solutions that are robust to variations of the environment and the capacity to scale-up to more complex versions of the problem. Consequently, differently from previous related works [8, 30–32, 38–39] that investigated the speed with which alternative method discover sufficiently good solutions, we compared alternative methods for the ability to: (i) generate high performing solutions, (ii) generate solutions that generalize in varying environmental conditions, (iii) scale up to complexified versions of the double-pole problem (i.e. a problem in which the agent needs to act on the basis of the state of the world at time t_-1_ and a problem in which the length and the mass of the shorter pole is increased from 1/10 to ½ the length and the mass of the long pole).

The obtained results show how the two new methods that we introduced in this paper, namely the Stochastic Steady State (SSS) and the Parallel Stochastic Hill Climber (PSHC) methods, and the Exponential Natural Evolutionary Strategy (xNES) method that has been introduced by [39, 54] largely outperform the other methods considered, namely the NeuroEvolution of Augmenting Topologies method (NEAT), the Neural Evolution through Cooperatively Coevolved Synapses (CoSyNE), and the Cartesian Genetic Programming of Artificial Neural Network (CGPANN) methods with respect to all criteria. Indeed, the first three methods are able to generate solutions displaying significantly better performance and generalization capabilities than the other three methods. The fact that the offset in performance between the former and the latter methods is greater in the delayed double-pole balancing problem than in the standard double-pole balancing problem demonstrates how the former method also scale-up better to more complex problems. Finally, the fact that best three methods quickly outperform the other methods indicates that the advantage in term of adaptive power is not gained at a cost of a reduced adaptive speed.

We minimized the effect of parameter setting by varying systematically the most important parameters and by considering the performance achieved with the best combination of parameters. This was possible only in part in the case of NEAT that requires to set many parameters. Consequently, clearly NEAT could produce better performance with different parameter values.

We hypothesize that the efficacy of the SSS and PSHC methods is due to their capacity to effectively retain adaptive traits, thanks to the utilization of a steady state selection, and to keep exploring new portions of the search space, thanks to the utilization of an effective method for regulating the selective pressure. The ability to avoid losing previously discovered adaptive traits is probably particularly important in hard problems that involve a high-dimensional search space and that require the accumulation and the preservation of many adaptive changes.

The high performance of the xNES could be explained by its ability to estimate and use local gradient information and by its tendency to explore promising directions of the search space. This could represent a disadvantage in problems affected by strong local minima. For an analysis of the performance of this method on the optimization of standard benchmark functions, see [39].

The comparison among the three best methods does not highlight a clear winner. The fact that the SSS and the xNES methods outperform the PSHC method in the case of the long double-pole problem, indicates that they have a greater ability to scale-up to more complex problems than the PSHC. An advantage of the SSS method with respect to the xNES method consists in the fact that it is simpler. On the other hand, the ability to estimate the local fitness gradient might enable the xNES method to outperform the SSS method in certain domains. This aspect that should be investigated in future research.

We also demonstrated that the utilization of randomly varying environmental conditions promotes the evolution of robust solutions and that the performance and the generalization abilities of the evolved agents are strongly influenced by the number of trials used for evaluating evolving individuals.

Finally, our results demonstrate that the methods that permit to evolve the topology and the connection weights of the network (i.e. NEAT and CGPANN) are outperformed by methods that operate with fixed topologies (i.e. PSHC, SSS, and xNES), at least in the case of the double-pole balancing problem domain. The PSHC* method introduced in this paper, instead, achieved performance that are comparable with those obtained by the best methods operating with fixed topology (i.e. PSHC, SSS, and xNES). Overall this indicates that whether or not the possibility to co-adapt the topology of the network can increase the adaptive power of neuroevolution still represents an open question. Future research in this area should clarify the characteristics of the domains in which the possibility to co-adapt the topology of the network can represent an advantage and should identify the way in which such advantage can be maximized.

Other areas that deserve farther research include the study of the characteristics of the evolving networks and the role of the environmental variations. The possibility to evolve network with heterogeneous neurons and topology (see for example [35, 55]) and/or networks provided with regulatory mechanisms (see for example [26]) represent promising research lines. Further research is required to verify whether these techniques can be embedded in methods that are competitive with respect to the state of the art. With regard to the role of environmental variations, we demonstrated the advantage of evaluating evolving agents in randomly varying environmental conditions and the advantage of exposing evolving agents to a limited number of variable conditions. Further research should investigate the effect of the rate of variations of the environmental conditions throughout generations and the effect of spatial versus temporal variations of the environmental conditions.

## Supporting information

S1 Fig Behavior exhibited by one agent during eight trials carried out in the Fixed Initial State condition. The data refer to one of the best agents evolved with the SSS method by using the Double-Poles Balancing Problem. Each curve displays the position of the cart during a corresponding trial.
(PDF)

S2 Fig Behavior exhibited by one agent during eight trials carried out in the Fixed Initial State condition. The data refer to one of the best agents evolved with the SSS method by using the Delayed Double-Poles Balancing Problem. Each curve displays the position of the cart during a corresponding trial.
(PDF)

S3 Fig Behavior exhibited by one agent during eight trials carried out in the Fixed Initial State condition. The data refer to one of the best agents evolved with the SSS method by using the Long Double-Poles Balancing Problem. Each curve displays the position of the cart during a corresponding trial.
(PDF)

S4 Fig Behavior exhibited by one agent during eight trials carried out in the Fixed Initial State condition. The data refer to one of the best agents evolved with the xNES method by using the Double-Poles Balancing Problem. Each curve displays the position of the cart during a corresponding trial.
(PDF)

S5 Fig Behavior exhibited by one agent during eight trials carried out in the Fixed Initial State condition. The data refer to one of the best agents evolved with the xNES method by using the Delayed Double-Poles Balancing Problem. Each curve displays the position of the cart during a corresponding trial.
(PDF)

S6 Fig Behavior exhibited by one agent during eight trials carried out in the Fixed Initial State condition. The data refer to one of the best agents evolved with the xNES method by using the Long Double-Poles Balancing Problem. Each curve display the position of the cart during a corresponding trial.
(PDF)

S1 Table Performance and generalization ability of neural network controllers evolved with the xNES method by varying the learning rate parameter. Each number indicates the average performance obtained during 30 replications of the experiment. Generalization refers to the average performance obtained by post-evaluating the evolved networks on 1000 trials during which the initial states of the cart have been set randomly. The numbers in square brackets indicate the number of trials in which the agents manage to maintain the poles balanced for the entire duration of the trial during the post-evaluation test. The best performance for each condition is indicated in bold.
(PDF)

S2 Table Performance and generalization ability of neural network controllers evolved with the NEAT method by varying the add neuron and population size parameters. Each number indicates the average performance obtained during 30 replications of the experiment. Generalization refers to the average performance obtained by post-evaluating the evolved networks on 1000 trials during which the initial states of the cart have been set randomly. The numbers in square brackets indicate the number of trials in which the agents manage to maintain the poles balanced for the entire duration of the trial during the post-evaluation test. The best performance for each condition is indicated in bold.
(PDF)

S3 Table Performance and generalization ability of neural network controllers evolved with the CGPANN method by varying the mutation rate and the incoming connection parameters. Each number indicates the average performance obtained during 30 replications of the experiment. Generalization refers to the average performance obtained by post-evaluating the evolved networks on 1000 trials during which the initial states of the cart have been set randomly. The numbers in square brackets indicate the number of trials in which the agents manage to maintain the poles balanced for the entire duration of the trial during the post-evaluation test. The best performance for each condition is indicated in bold.
(PDF)

S4 Table Performance and generalization ability of neural network controllers evolved with the CoSyNE method by varying the mutation rate and the subpopulation parameters. Each number indicates the average performance obtained during 30 replications of the experiment. Generalization refers to the average performance obtained by post-evaluating the evolved networks on 1000 trials during which the initial states of the cart have been set randomly. The numbers in square brackets indicate the number of trials in which the agents manage to maintain the poles balanced for the entire duration of the trial during the post-evaluation test. The best performance for each condition is indicated in bold.
(PDF)

S5 Table Performance and generalization ability of neural network controllers evolved with the PSHC method by varying the mutation rate and the stochasticity parameters. Each number indicates the average performance obtained during 30 replications of the experiment. Generalization refers to the average performance obtained by post-evaluating the evolved networks on 1000 trials during which the initial states of the cart have been set randomly. The numbers in square brackets indicate the number of trials in which the agents manage to maintain the poles balanced for the entire duration of the trial during the post-evaluation test. The best performance for each condition is indicated in bold. For all the experiment the annealing length is set to 200 steps.
(PDF)

S1 File Modifications of source code of NEAT. (ZIP)

S2 File Modifications of source code of CoSyNE. (ZIP)

S3 File Source code of FARSA. (ZIP)

S4 File Source code of CGPANN. (ZIP)

S5 File Source code of xNES. (ZIP)

S6 File Source code of SSS. (ZIP)

S7 File Source code of PSHC. (ZIP)
