# Supplementary figures and images for: Maximizing adaptive power in neuroevolution
Source: PLoS One. 2018 Jul 18;13(7):e0198788. doi: 10.1371/journal.pone.0198788 (PMC6051599; doi:10.1371/journal.pone.0198788)

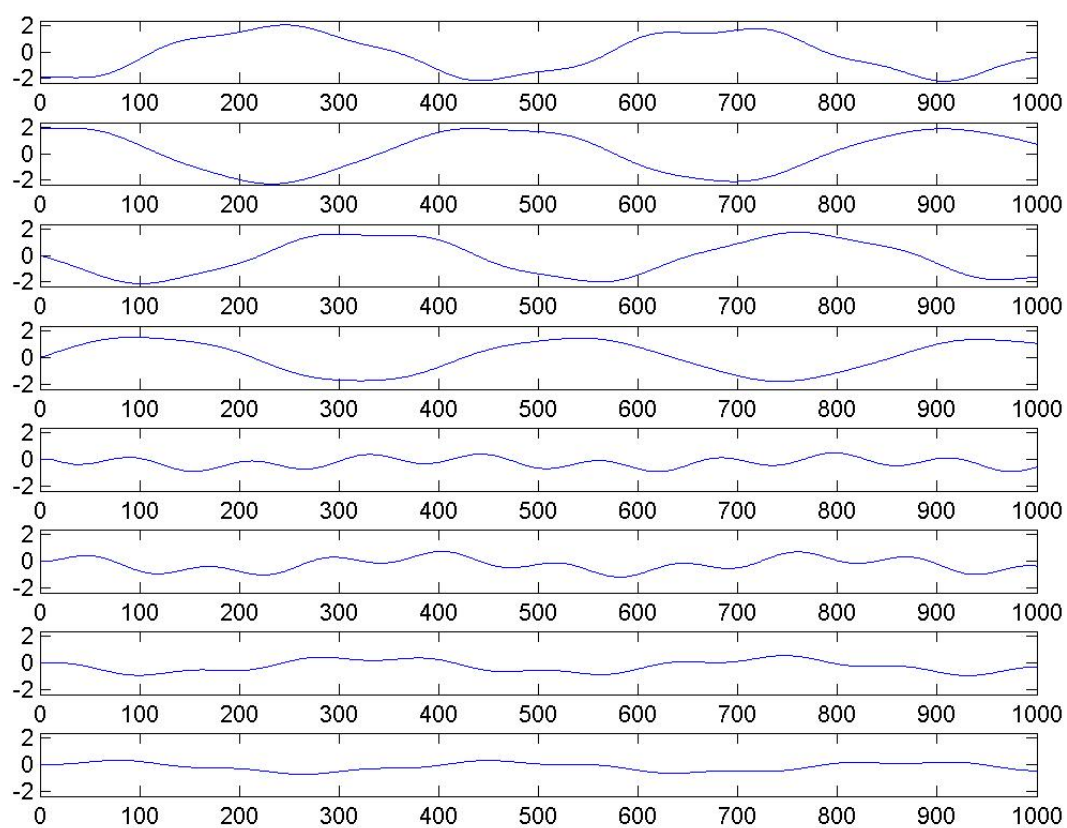

Supplement: S1 Fig — The data refer to one of the best agents evolved with the SSS method by using the Double-Poles Balancing Problem. Each curve displays the position of the cart during a corresponding trial. (PDF) [file pone.0198788.s001.pdf]

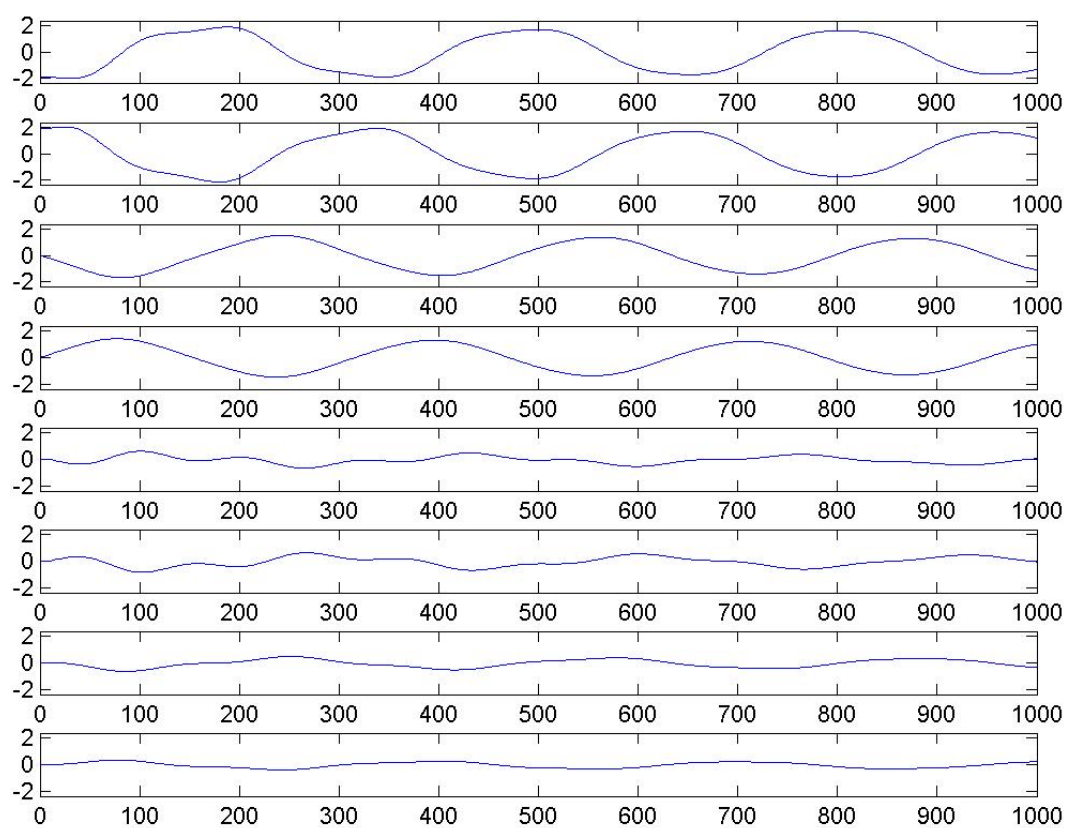

Supplement: S2 Fig — The data refer to one of the best agents evolved with the SSS method by using the Delayed Double-Poles Balancing Problem. Each curve displays the position of the cart during a corresponding trial. (PDF) [file pone.0198788.s002.pdf]

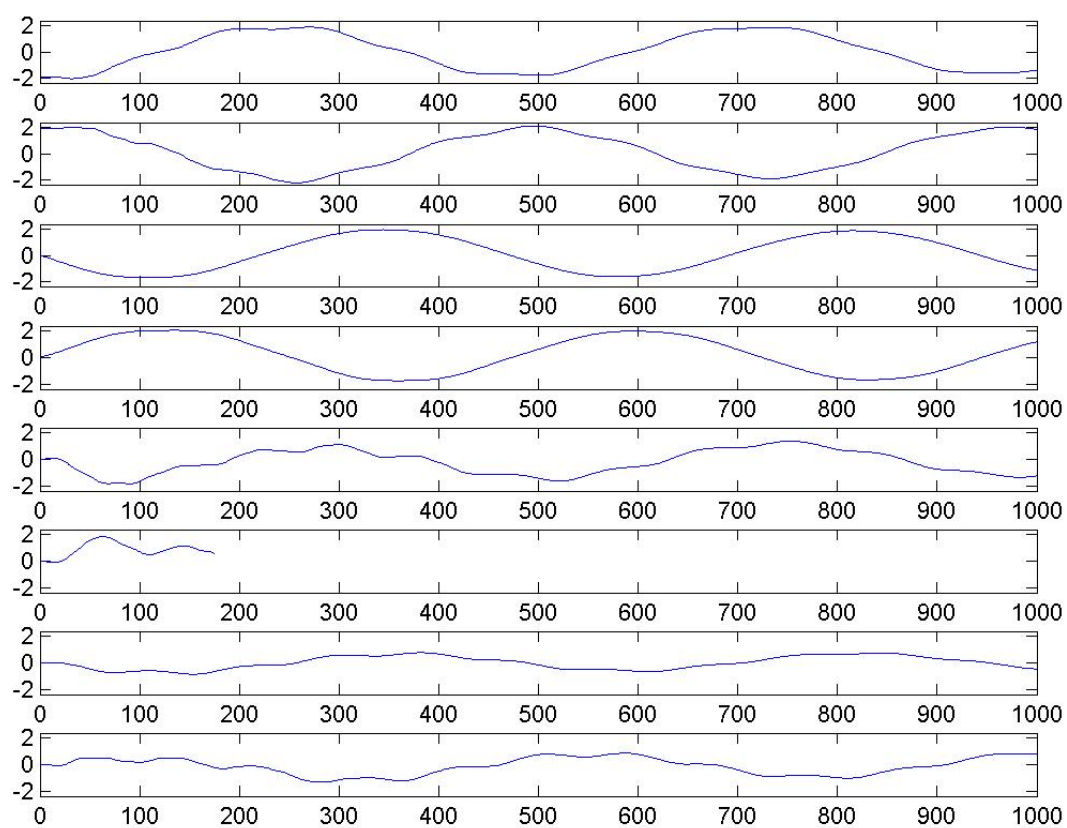

Supplement: S3 Fig — The data refer to one of the best agents evolved with the SSS method by using the Long Double-Poles Balancing Problem. Each curve displays the position of the cart during a corresponding trial. (PDF) [file pone.0198788.s003.pdf]

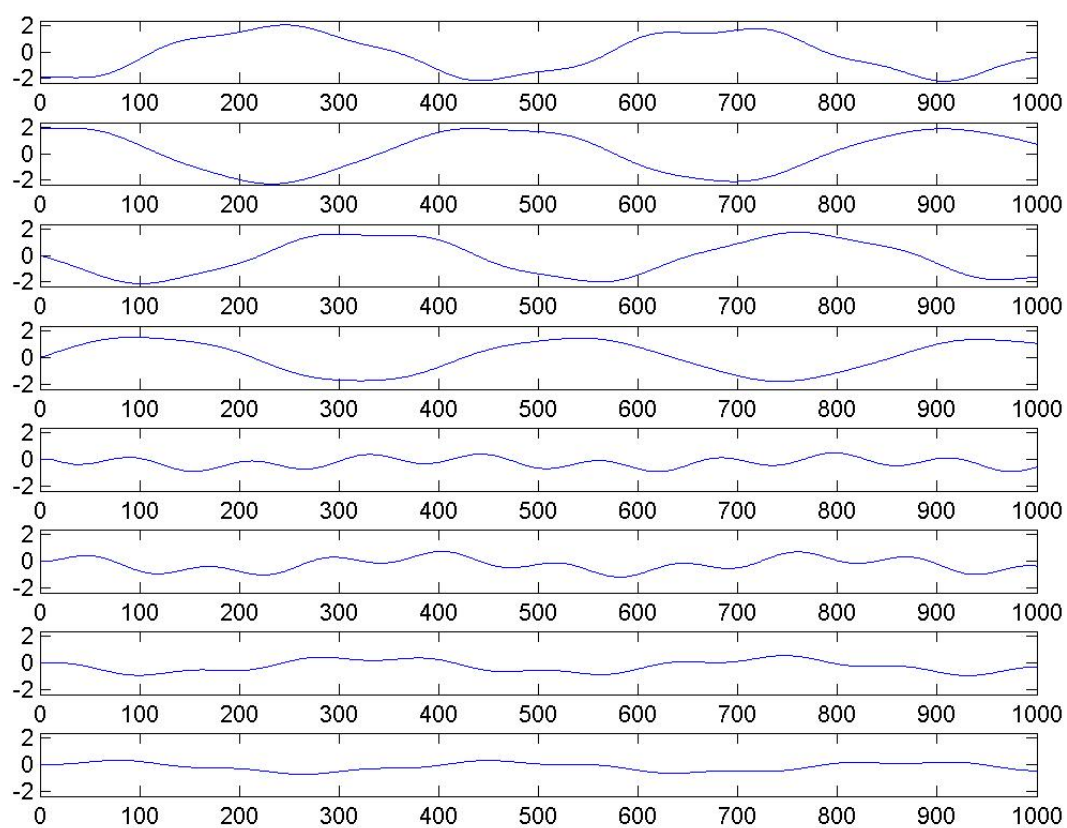

Supplement: S4 Fig — The data refer to one of the best agents evolved with the xNES method by using the Double-Poles Balancing Problem. Each curve displays the position of the cart during a corresponding trial. (PDF) [file pone.0198788.s004.pdf]

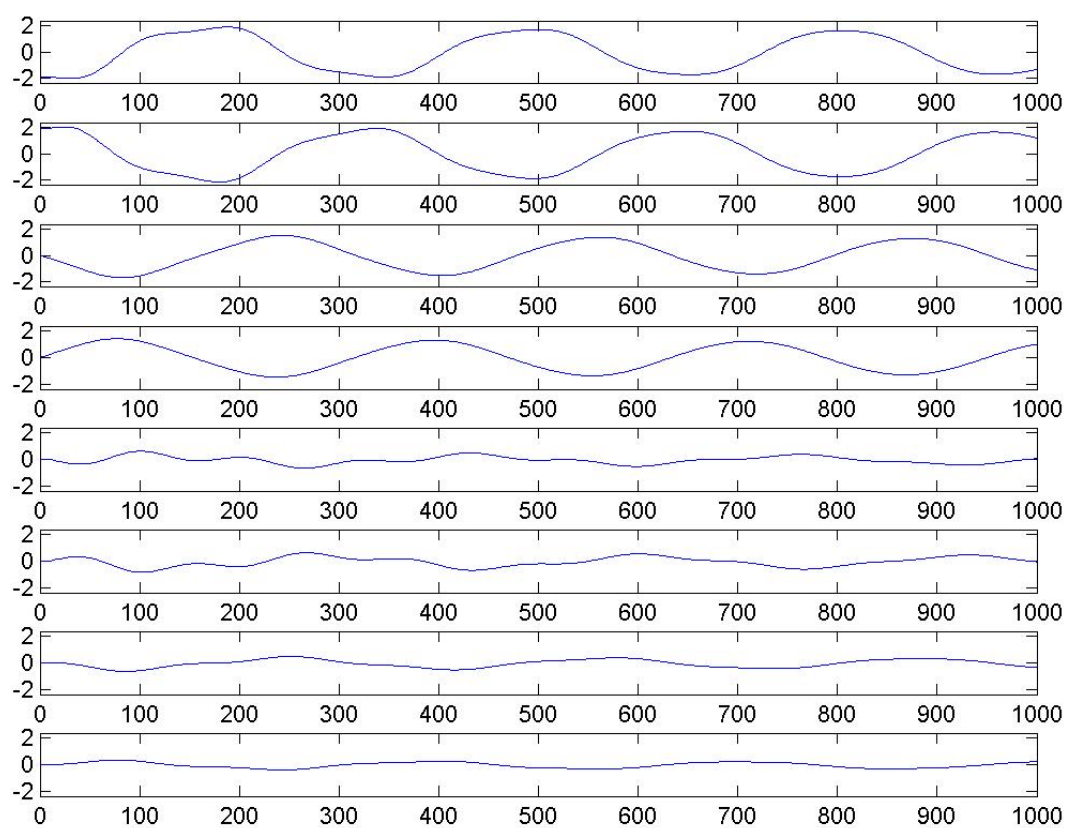

Supplement: S5 Fig — The data refer to one of the best agents evolved with the xNES method by using the Delayed Double-Poles Balancing Problem. Each curve displays the position of the cart during a corresponding trial. (PDF) [file pone.0198788.s005.pdf]

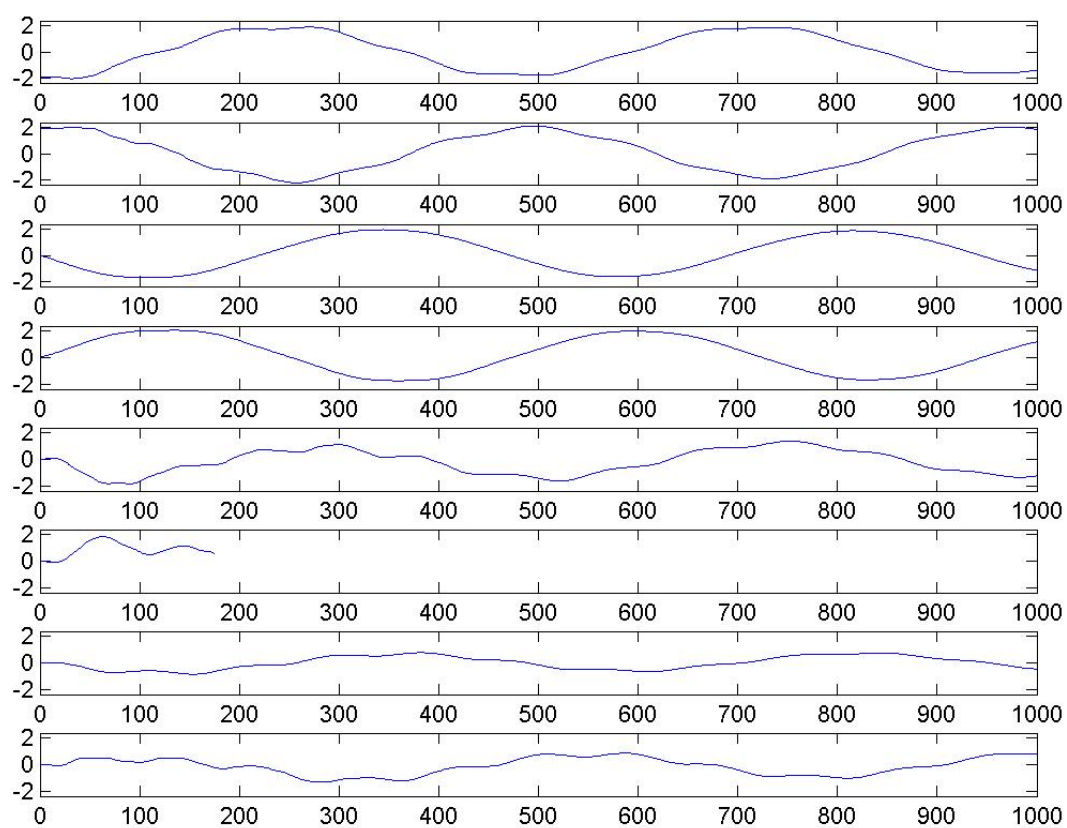

Supplement: S6 Fig — The data refer to one of the best agents evolved with the xNES method by using the Long Double-Poles Balancing Problem. Each curve display the position of the cart during a corresponding trial. (PDF) [file pone.0198788.s006.pdf]

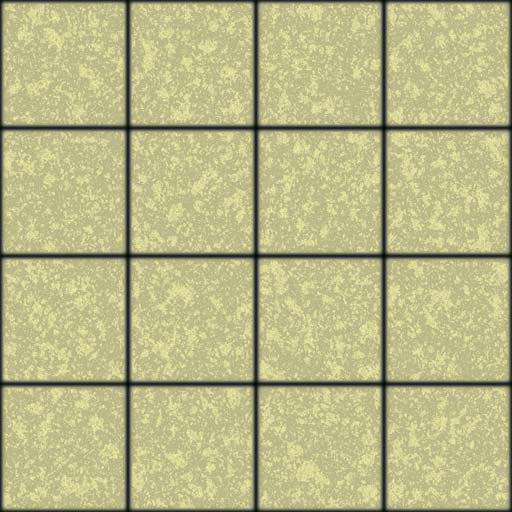

Supplement: S3 File — (ZIP) [file pone.0198788.s014.zip › farsa-1.4.5/worldsim/textures/tiles/16tile08.jpg]

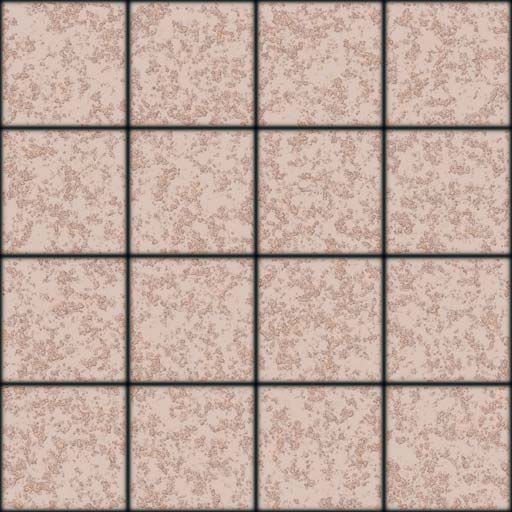

Supplement: S3 File — (ZIP) [file pone.0198788.s014.zip › farsa-1.4.5/worldsim/textures/tiles/16tile07.jpg]

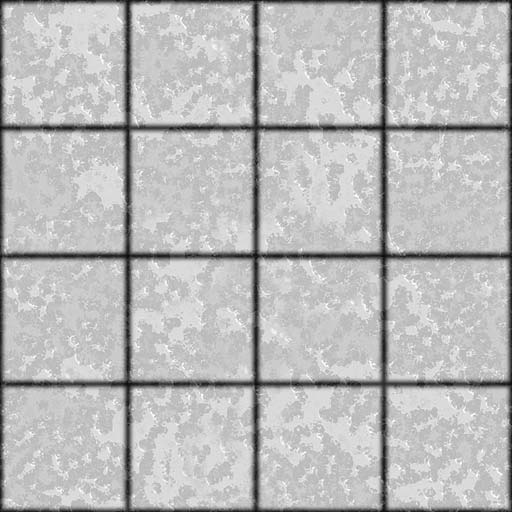

Supplement: S3 File — (ZIP) [file pone.0198788.s014.zip › farsa-1.4.5/worldsim/textures/tiles/16tile12.jpg]

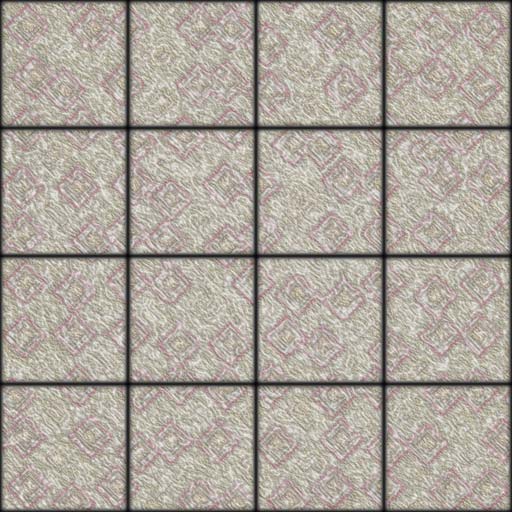

Supplement: S3 File — (ZIP) [file pone.0198788.s014.zip › farsa-1.4.5/worldsim/textures/tiles/16tile06.jpg]

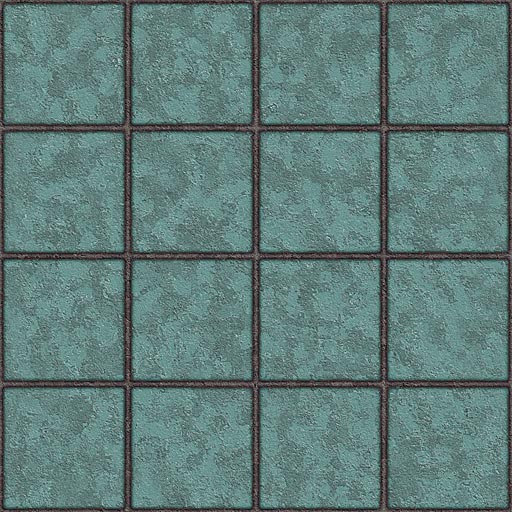

Supplement: S3 File — (ZIP) [file pone.0198788.s014.zip › farsa-1.4.5/worldsim/textures/tiles/16tile01.jpg]

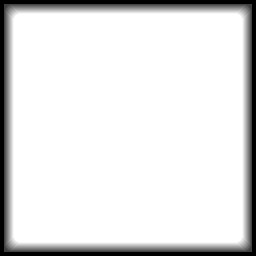

Supplement: S3 File — (ZIP) [file pone.0198788.s014.zip › farsa-1.4.5/worldsim/textures/tiles/tilebump.jpg]

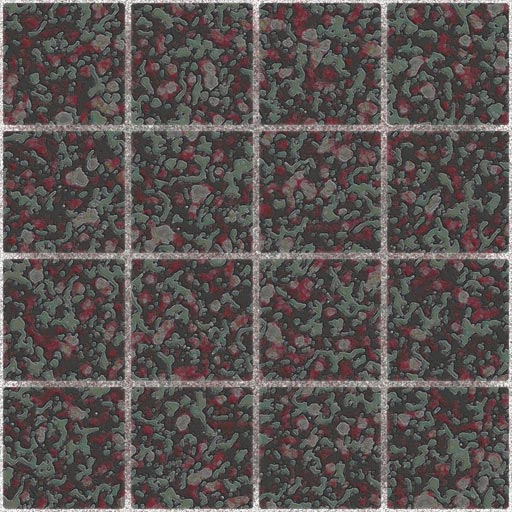

Supplement: S3 File — (ZIP) [file pone.0198788.s014.zip › farsa-1.4.5/worldsim/textures/tiles/16tile04.jpg]

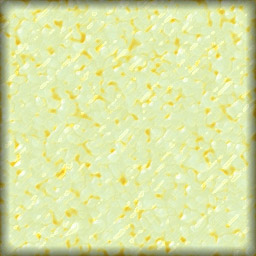

Supplement: S3 File — (ZIP) [file pone.0198788.s014.zip › farsa-1.4.5/worldsim/textures/tiles/tile03.jpg]

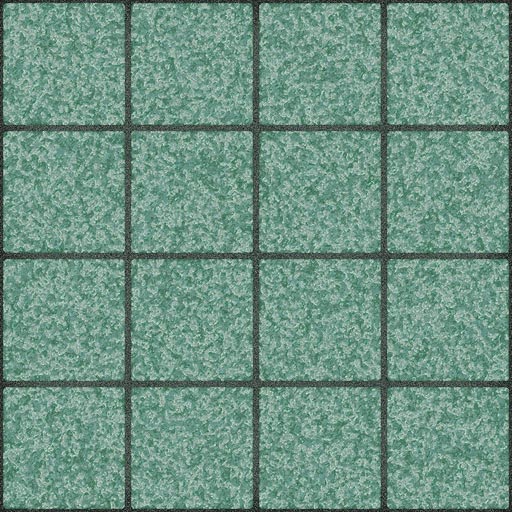

Supplement: S3 File — (ZIP) [file pone.0198788.s014.zip › farsa-1.4.5/worldsim/textures/tiles/16tile05.jpg]

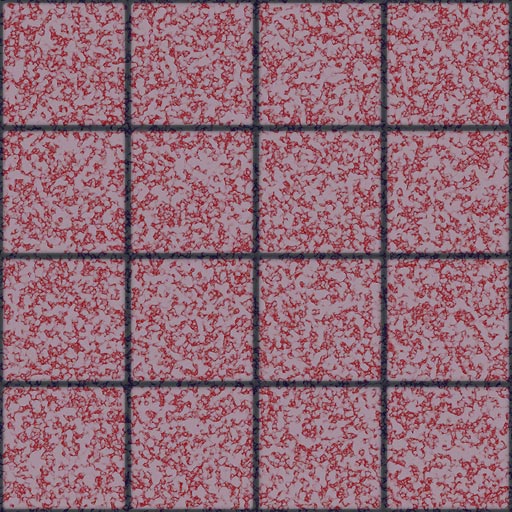

Supplement: S3 File — (ZIP) [file pone.0198788.s014.zip › farsa-1.4.5/worldsim/textures/tiles/16tile02.jpg]

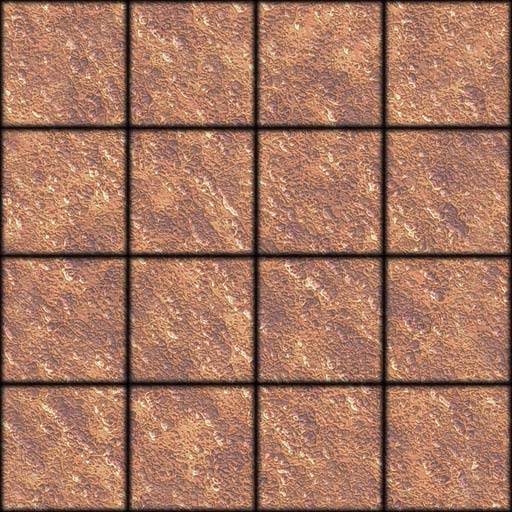

Supplement: S3 File — (ZIP) [file pone.0198788.s014.zip › farsa-1.4.5/worldsim/textures/tiles/16tile13.jpg]

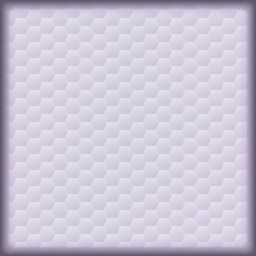

Supplement: S3 File — (ZIP) [file pone.0198788.s014.zip › farsa-1.4.5/worldsim/textures/tiles/tile05.jpg]

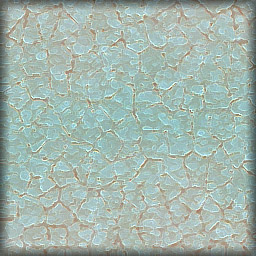

Supplement: S3 File — (ZIP) [file pone.0198788.s014.zip › farsa-1.4.5/worldsim/textures/tiles/tile04.jpg]

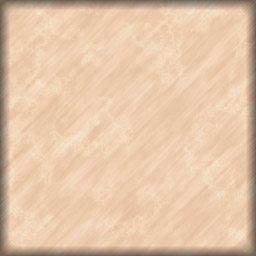

Supplement: S3 File — (ZIP) [file pone.0198788.s014.zip › farsa-1.4.5/worldsim/textures/tiles/tile01.jpg]

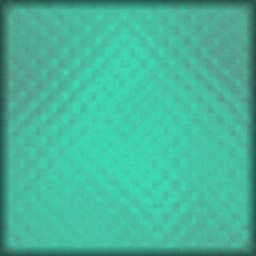

Supplement: S3 File — (ZIP) [file pone.0198788.s014.zip › farsa-1.4.5/worldsim/textures/tiles/tile02.jpg]

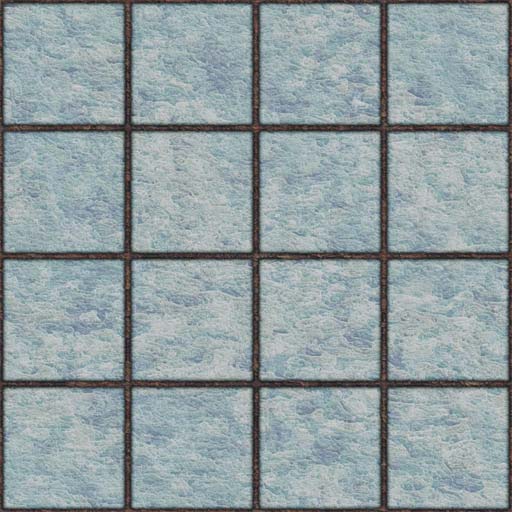

Supplement: S3 File — (ZIP) [file pone.0198788.s014.zip › farsa-1.4.5/worldsim/textures/tiles/16tile11.jpg]

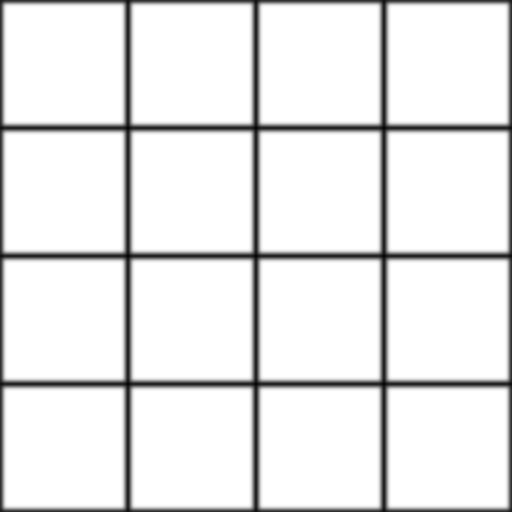

Supplement: S3 File — (ZIP) [file pone.0198788.s014.zip › farsa-1.4.5/worldsim/textures/tiles/16tile-B.jpg]

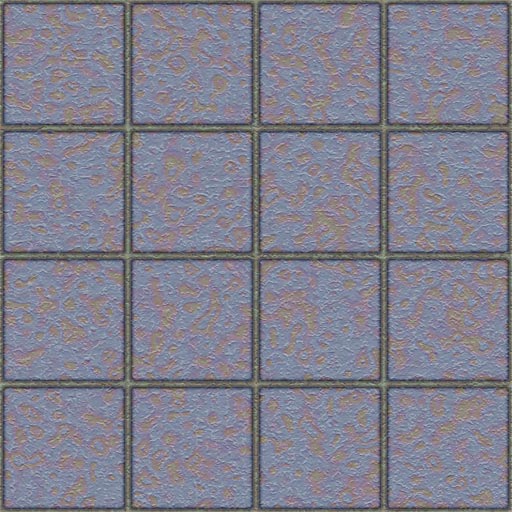

Supplement: S3 File — (ZIP) [file pone.0198788.s014.zip › farsa-1.4.5/worldsim/textures/tiles/16tile03.jpg]

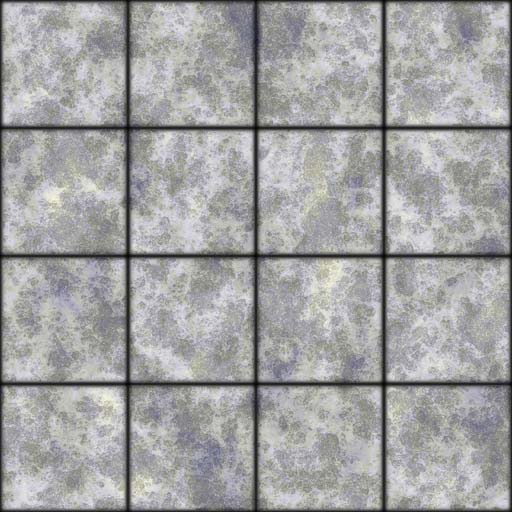

Supplement: S3 File — (ZIP) [file pone.0198788.s014.zip › farsa-1.4.5/worldsim/textures/tiles/16tile10.jpg]

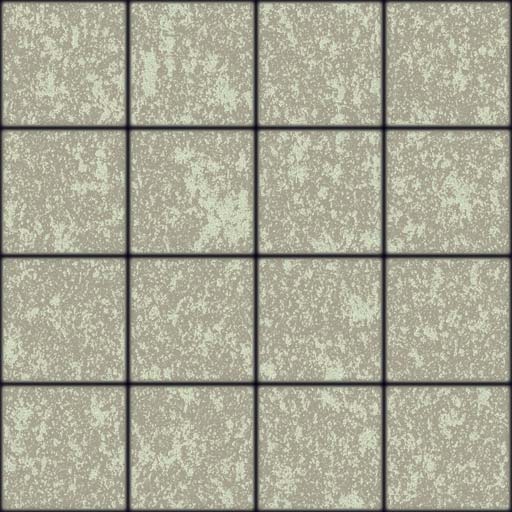

Supplement: S3 File — (ZIP) [file pone.0198788.s014.zip › farsa-1.4.5/worldsim/textures/tiles/16tile09.jpg]

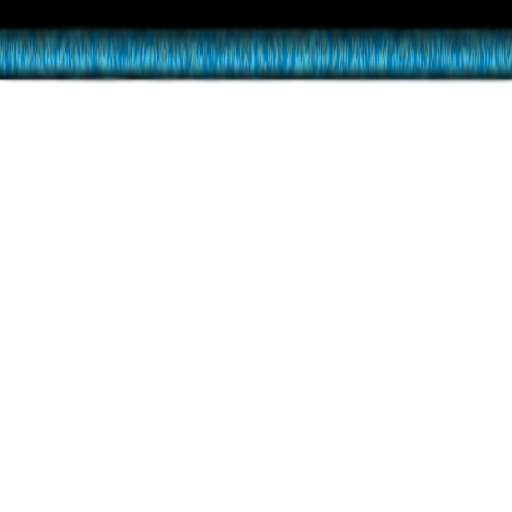

Supplement: S3 File — (ZIP) [file pone.0198788.s014.zip › farsa-1.4.5/worldsim/textures/covers/eyep2_b.jpg]

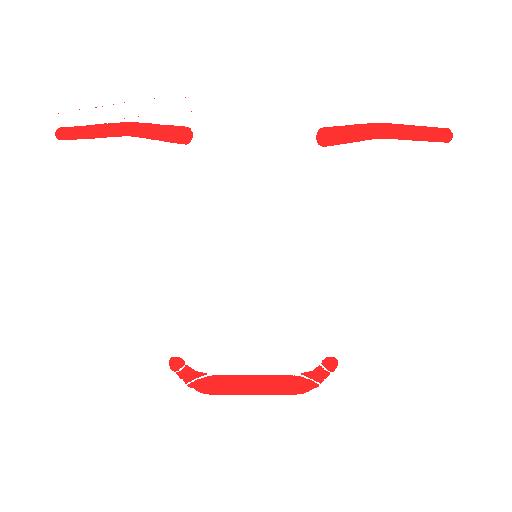

Supplement: S3 File — (ZIP) [file pone.0198788.s014.zip › farsa-1.4.5/worldsim/textures/covers/face.jpg]

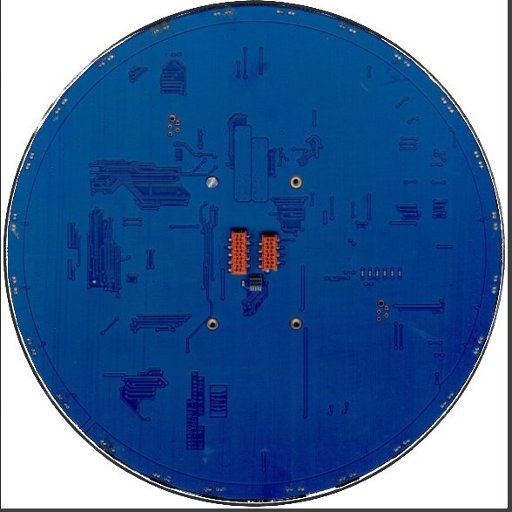

Supplement: S3 File — (ZIP) [file pone.0198788.s014.zip › farsa-1.4.5/worldsim/textures/covers/marxbot_12leds.jpg]

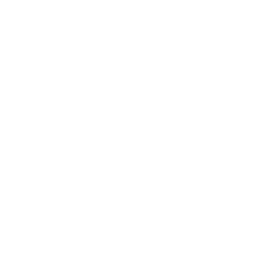

Supplement: S3 File — (ZIP) [file pone.0198788.s014.zip › farsa-1.4.5/worldsim/textures/white.jpg]

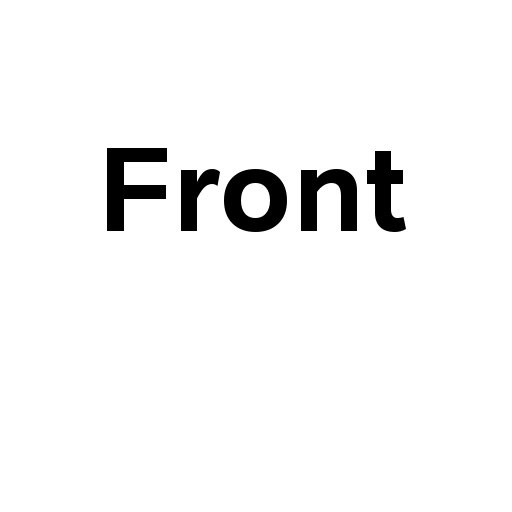

Supplement: S3 File — (ZIP) [file pone.0198788.s014.zip › farsa-1.4.5/worldsim/textures/skybox/sb_front.jpg]

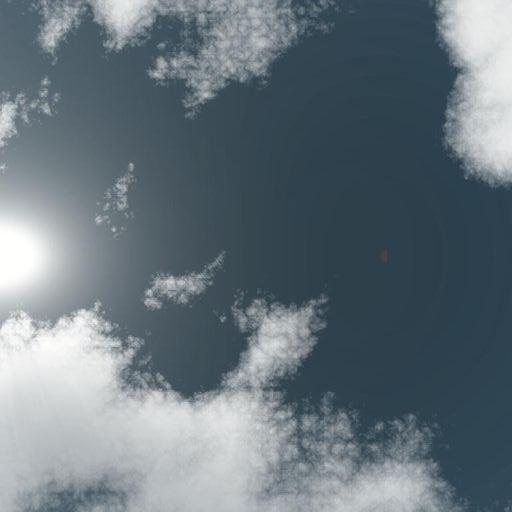

Supplement: S3 File — (ZIP) [file pone.0198788.s014.zip › farsa-1.4.5/worldsim/textures/skybox/sb2_top.jpg]

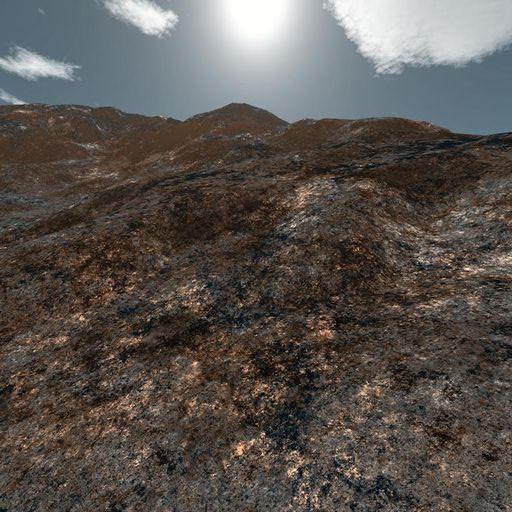

Supplement: S3 File — (ZIP) [file pone.0198788.s014.zip › farsa-1.4.5/worldsim/textures/skybox/sb2_left.jpg]

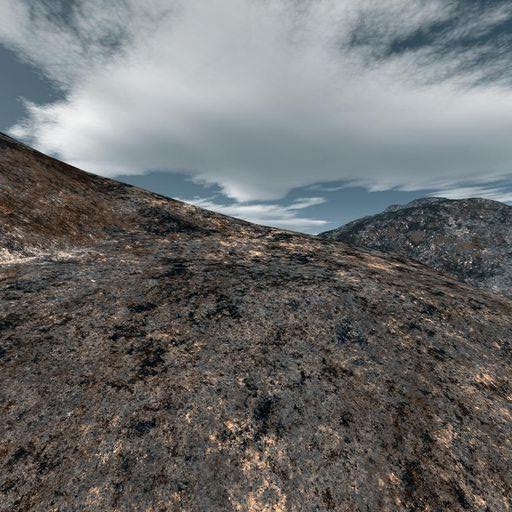

Supplement: S3 File — (ZIP) [file pone.0198788.s014.zip › farsa-1.4.5/worldsim/textures/skybox/sb2_front.jpg]

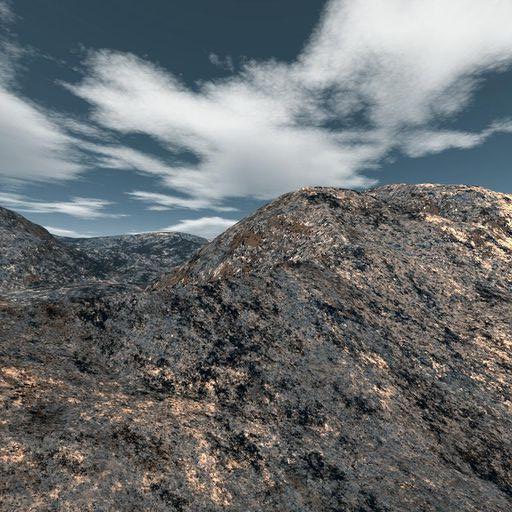

Supplement: S3 File — (ZIP) [file pone.0198788.s014.zip › farsa-1.4.5/worldsim/textures/skybox/sb2_right.jpg]

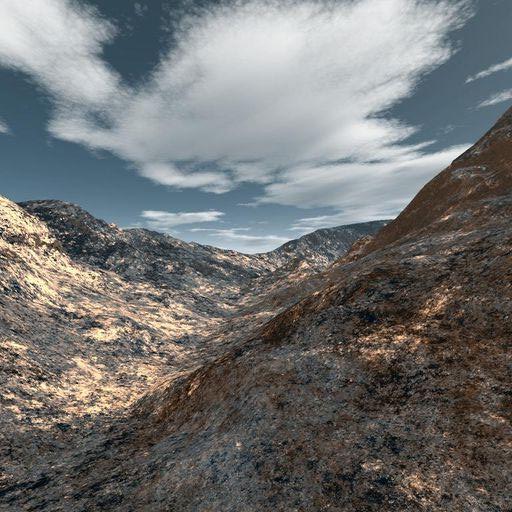

Supplement: S3 File — (ZIP) [file pone.0198788.s014.zip › farsa-1.4.5/worldsim/textures/skybox/sb2_back.jpg]

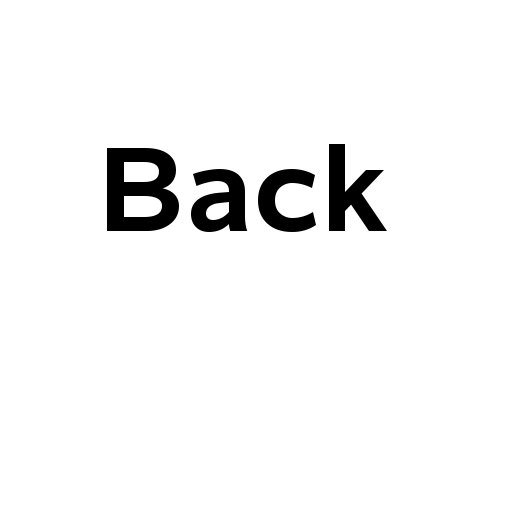

Supplement: S3 File — (ZIP) [file pone.0198788.s014.zip › farsa-1.4.5/worldsim/textures/skybox/sb_back.jpg]

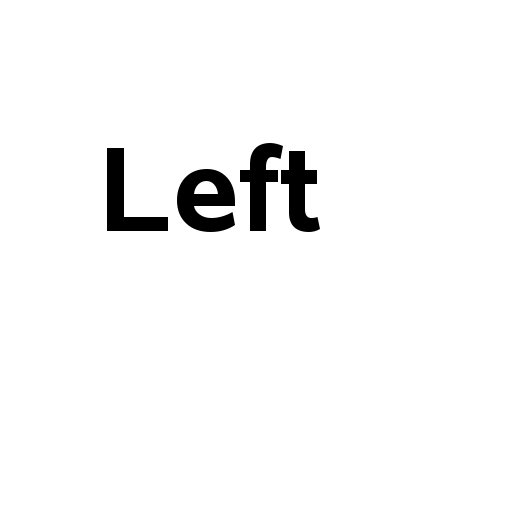

Supplement: S3 File — (ZIP) [file pone.0198788.s014.zip › farsa-1.4.5/worldsim/textures/skybox/sb_left.jpg]

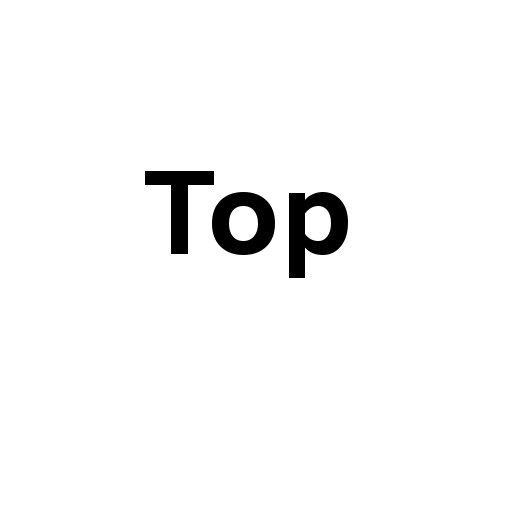

Supplement: S3 File — (ZIP) [file pone.0198788.s014.zip › farsa-1.4.5/worldsim/textures/skybox/sb_top.jpg]

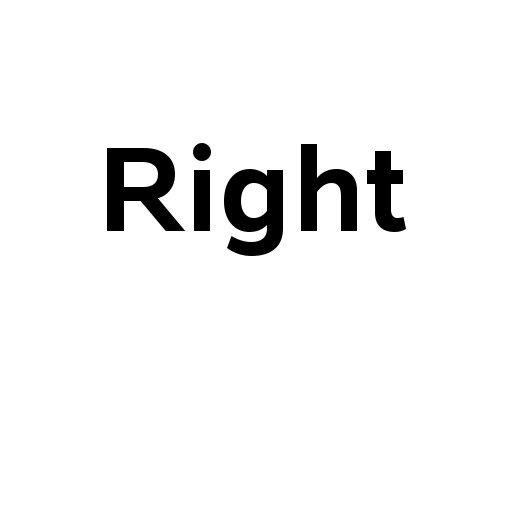

Supplement: S3 File — (ZIP) [file pone.0198788.s014.zip › farsa-1.4.5/worldsim/textures/skybox/sb_right.jpg]

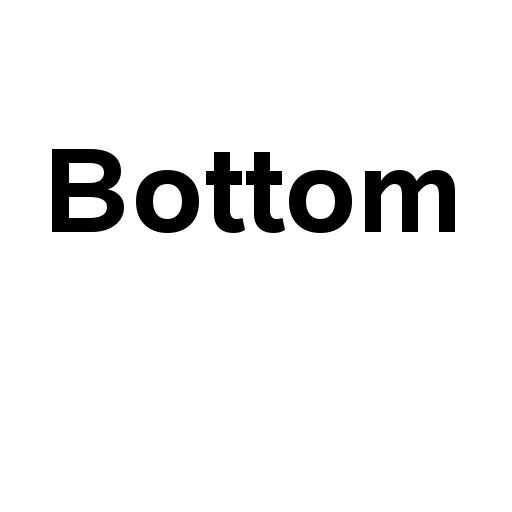

Supplement: S3 File — (ZIP) [file pone.0198788.s014.zip › farsa-1.4.5/worldsim/textures/skybox/sb_bottom.jpg]

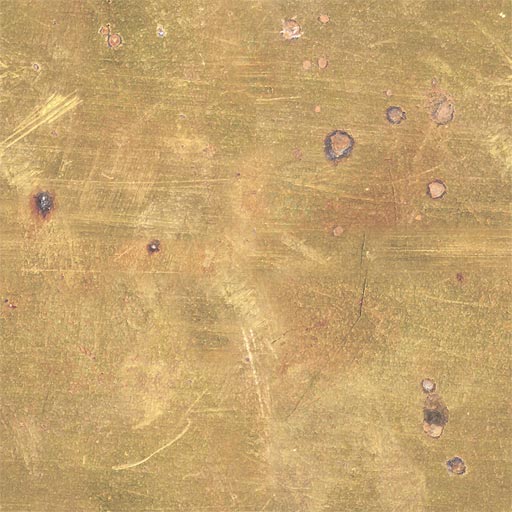

Supplement: S3 File — (ZIP) [file pone.0198788.s014.zip › farsa-1.4.5/worldsim/textures/metal/brass01.jpg]

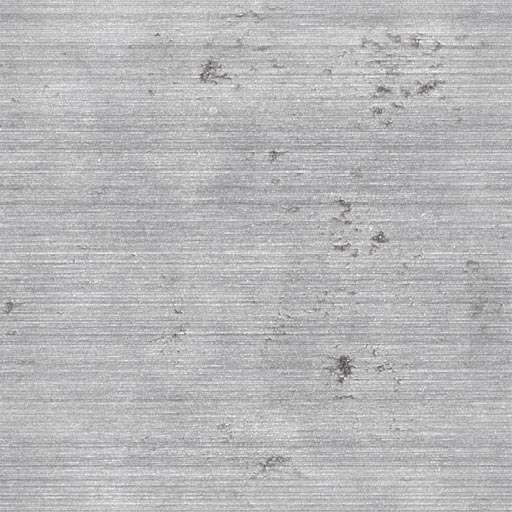

Supplement: S3 File — (ZIP) [file pone.0198788.s014.zip › farsa-1.4.5/worldsim/textures/metal/iron05.jpg]

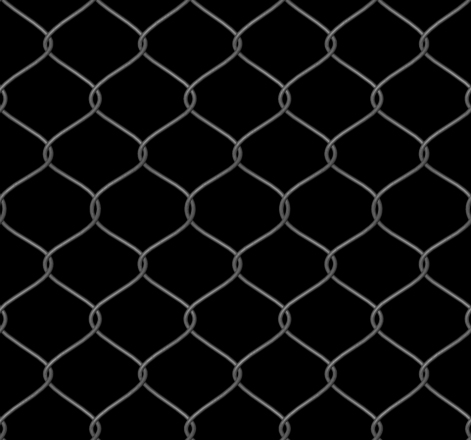

Supplement: S3 File — (ZIP) [file pone.0198788.s014.zip › farsa-1.4.5/worldsim/textures/metal/zaun_tex.jpg]

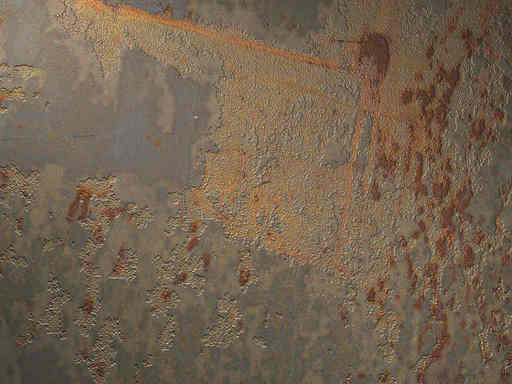

Supplement: S3 File — (ZIP) [file pone.0198788.s014.zip › farsa-1.4.5/worldsim/textures/metal/metbgr02.jpg]

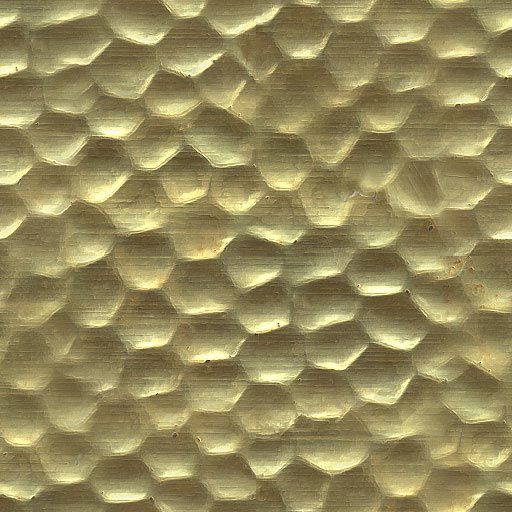

Supplement: S3 File — (ZIP) [file pone.0198788.s014.zip › farsa-1.4.5/worldsim/textures/metal/hambrass.jpg]

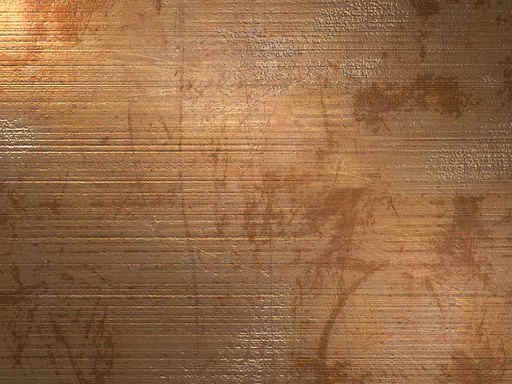

Supplement: S3 File — (ZIP) [file pone.0198788.s014.zip › farsa-1.4.5/worldsim/textures/metal/metbgr03.jpg]

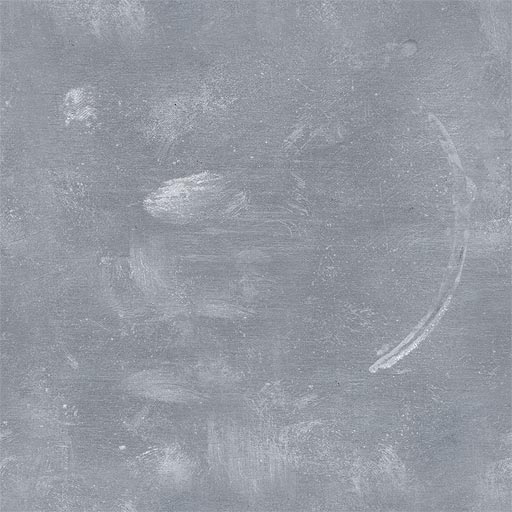

Supplement: S3 File — (ZIP) [file pone.0198788.s014.zip › farsa-1.4.5/worldsim/textures/metal/zinc02.jpg]

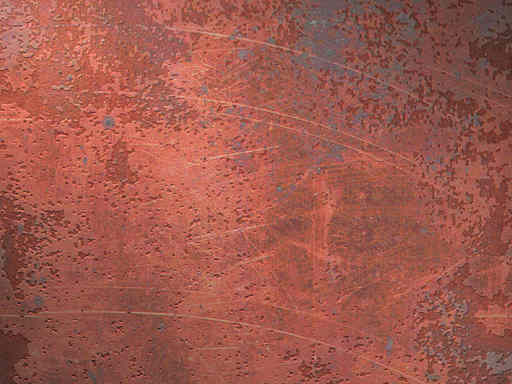

Supplement: S3 File — (ZIP) [file pone.0198788.s014.zip › farsa-1.4.5/worldsim/textures/metal/metbgr04.jpg]

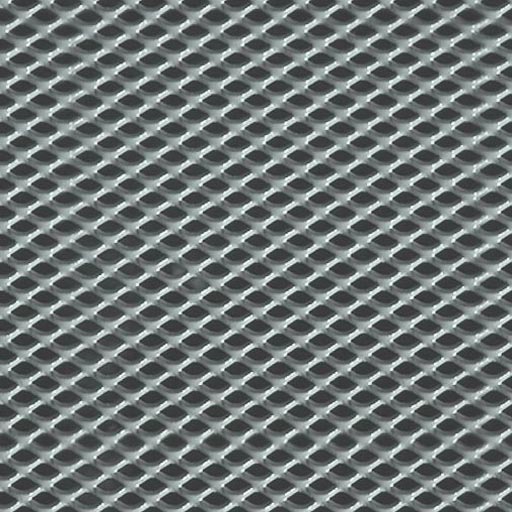

Supplement: S3 File — (ZIP) [file pone.0198788.s014.zip › farsa-1.4.5/worldsim/textures/metal/m_mesh01.jpg]

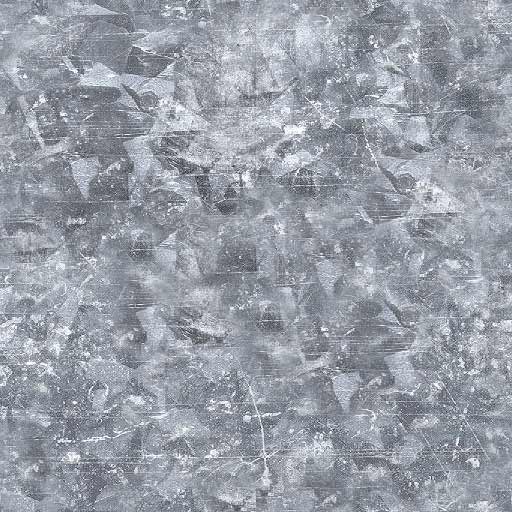

Supplement: S3 File — (ZIP) [file pone.0198788.s014.zip › farsa-1.4.5/worldsim/textures/metal/zinc01.jpg]

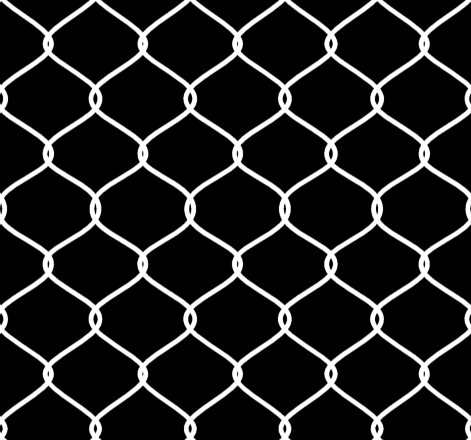

Supplement: S3 File — (ZIP) [file pone.0198788.s014.zip › farsa-1.4.5/worldsim/textures/metal/zaun_a.jpg]

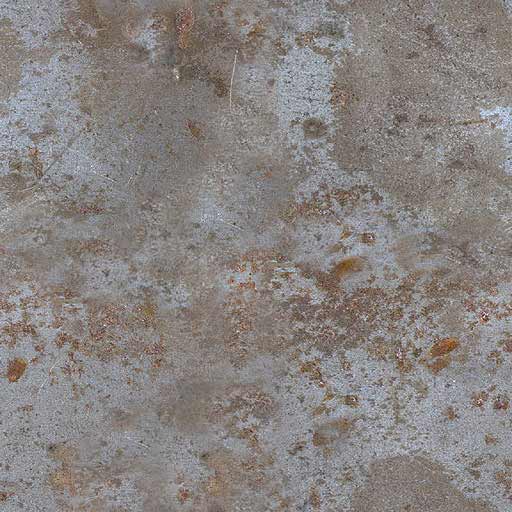

Supplement: S3 File — (ZIP) [file pone.0198788.s014.zip › farsa-1.4.5/worldsim/textures/metal/iron02.jpg]

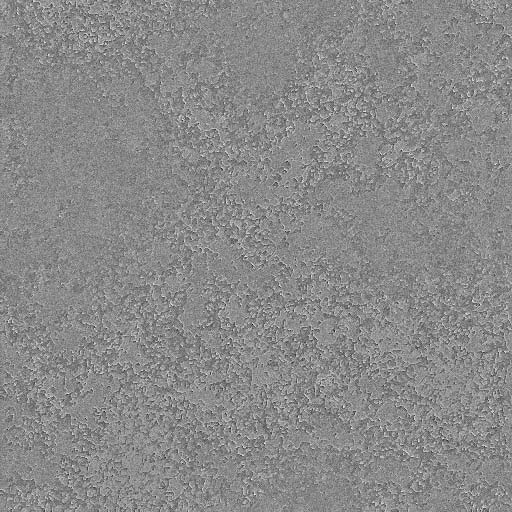

Supplement: S3 File — (ZIP) [file pone.0198788.s014.zip › farsa-1.4.5/worldsim/textures/metal/iron04.jpg]

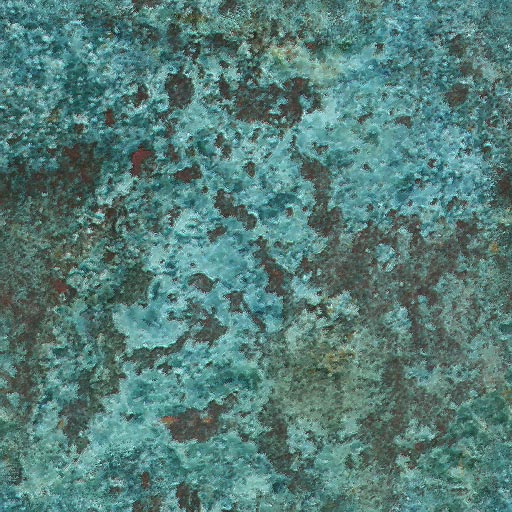

Supplement: S3 File — (ZIP) [file pone.0198788.s014.zip › farsa-1.4.5/worldsim/textures/metal/verdig01.jpg]

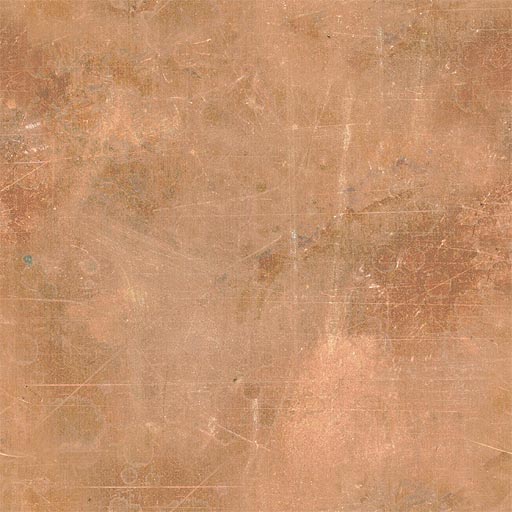

Supplement: S3 File — (ZIP) [file pone.0198788.s014.zip › farsa-1.4.5/worldsim/textures/metal/copper02.jpg]

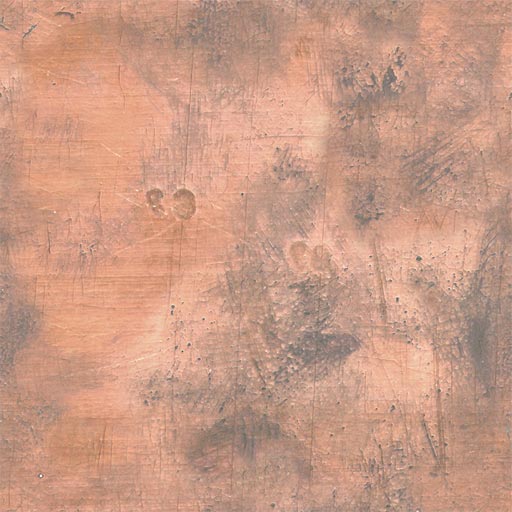

Supplement: S3 File — (ZIP) [file pone.0198788.s014.zip › farsa-1.4.5/worldsim/textures/metal/copper01.jpg]

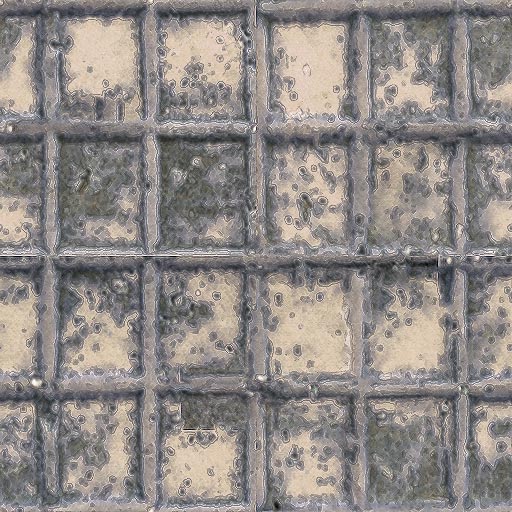

Supplement: S3 File — (ZIP) [file pone.0198788.s014.zip › farsa-1.4.5/worldsim/textures/metal/plate01.jpg]

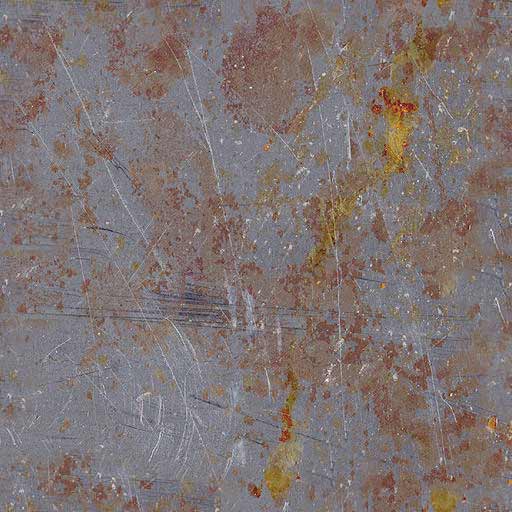

Supplement: S3 File — (ZIP) [file pone.0198788.s014.zip › farsa-1.4.5/worldsim/textures/metal/iron03.jpg]

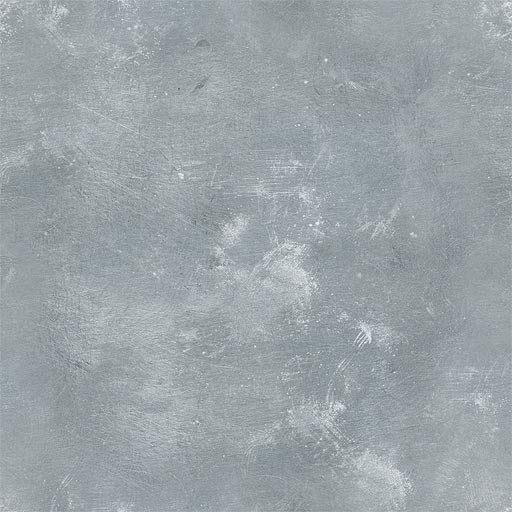

Supplement: S3 File — (ZIP) [file pone.0198788.s014.zip › farsa-1.4.5/worldsim/textures/metal/zinc03.jpg]

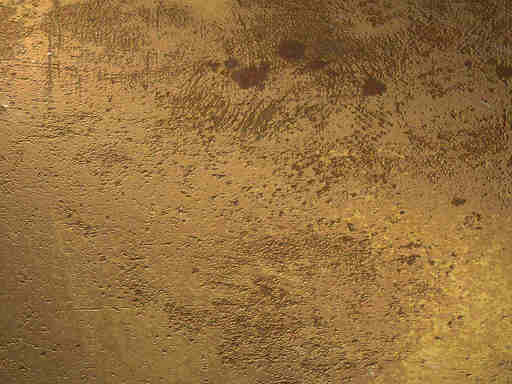

Supplement: S3 File — (ZIP) [file pone.0198788.s014.zip › farsa-1.4.5/worldsim/textures/metal/metbgr01.jpg]

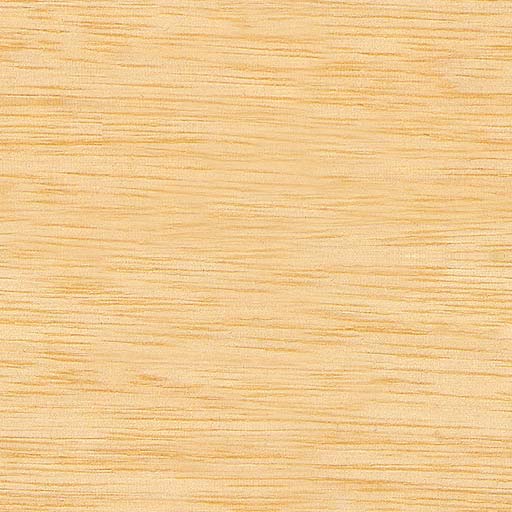

Supplement: S3 File — (ZIP) [file pone.0198788.s014.zip › farsa-1.4.5/worldsim/textures/metal/limba.jpg]

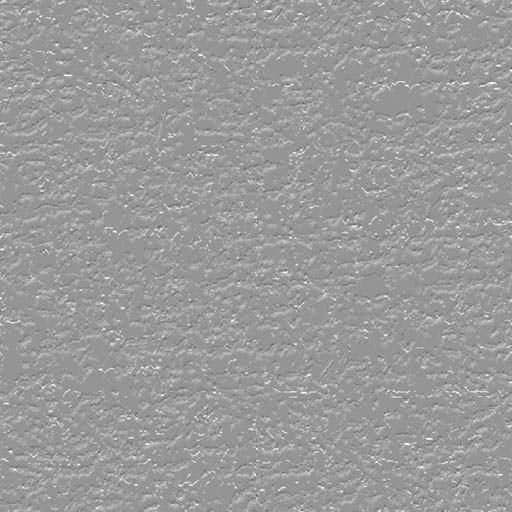

Supplement: S3 File — (ZIP) [file pone.0198788.s014.zip › farsa-1.4.5/worldsim/textures/metal/iron01.jpg]

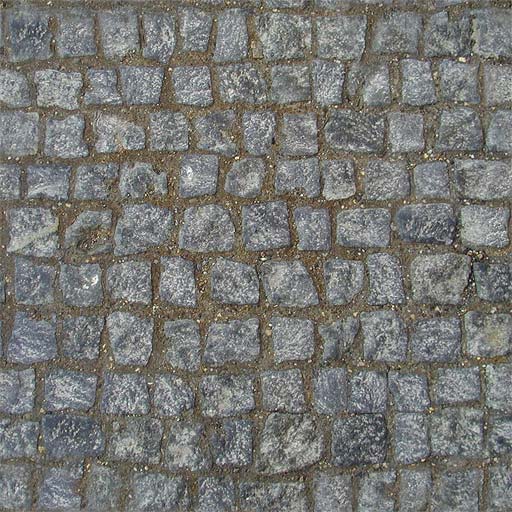

Supplement: S3 File — (ZIP) [file pone.0198788.s014.zip › farsa-1.4.5/worldsim/textures/ground/cobbles01.jpg]

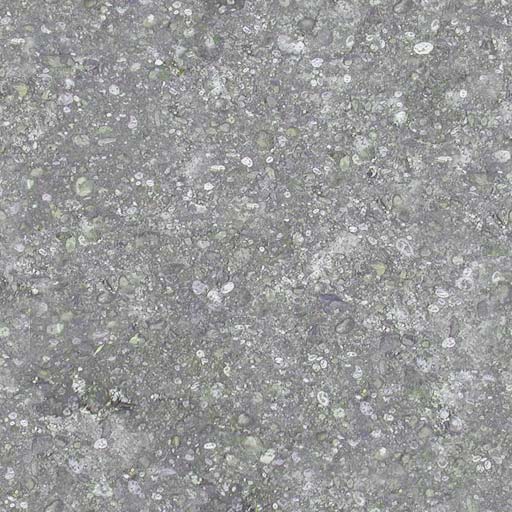

Supplement: S3 File — (ZIP) [file pone.0198788.s014.zip › farsa-1.4.5/worldsim/textures/ground/asph01.jpg]

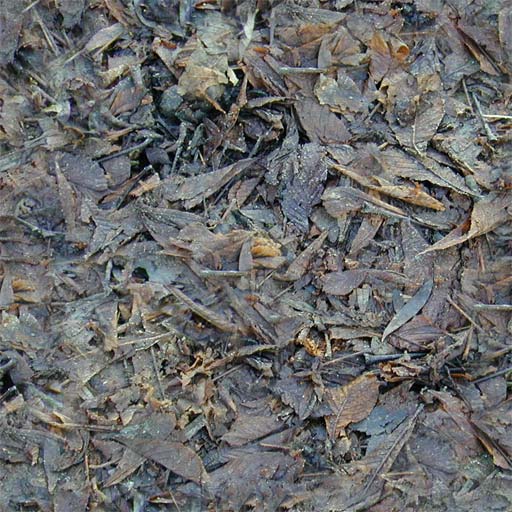

Supplement: S3 File — (ZIP) [file pone.0198788.s014.zip › farsa-1.4.5/worldsim/textures/ground/ground05.jpg]

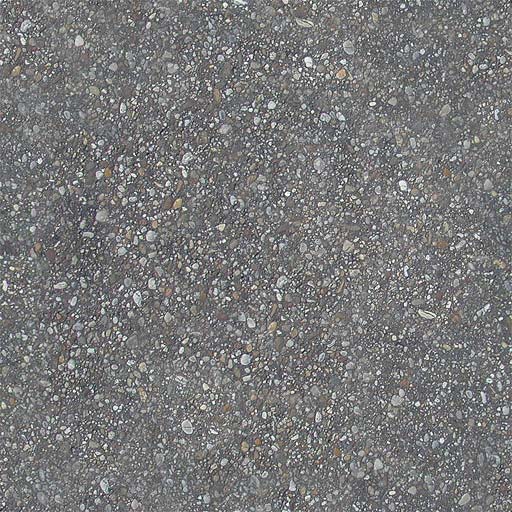

Supplement: S3 File — (ZIP) [file pone.0198788.s014.zip › farsa-1.4.5/worldsim/textures/ground/asph03.jpg]

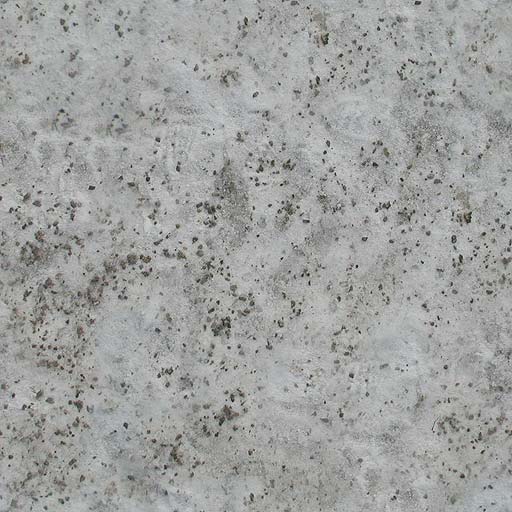

Supplement: S3 File — (ZIP) [file pone.0198788.s014.zip › farsa-1.4.5/worldsim/textures/ground/snowy03.jpg]

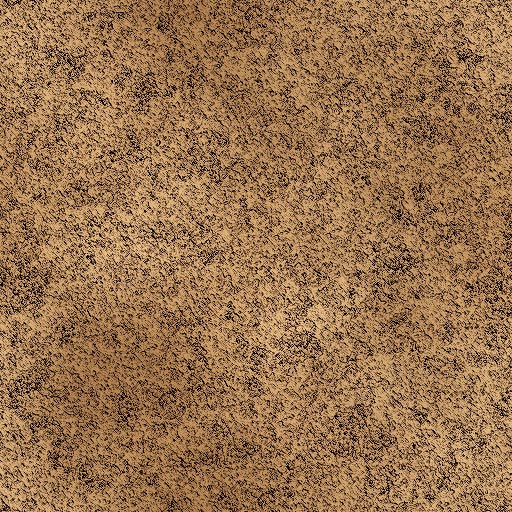

Supplement: S3 File — (ZIP) [file pone.0198788.s014.zip › farsa-1.4.5/worldsim/textures/ground/mud01.jpg]

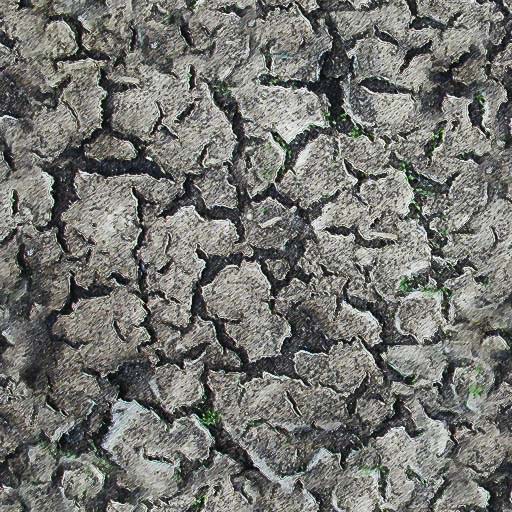

Supplement: S3 File — (ZIP) [file pone.0198788.s014.zip › farsa-1.4.5/worldsim/textures/ground/dry_mud.jpg]

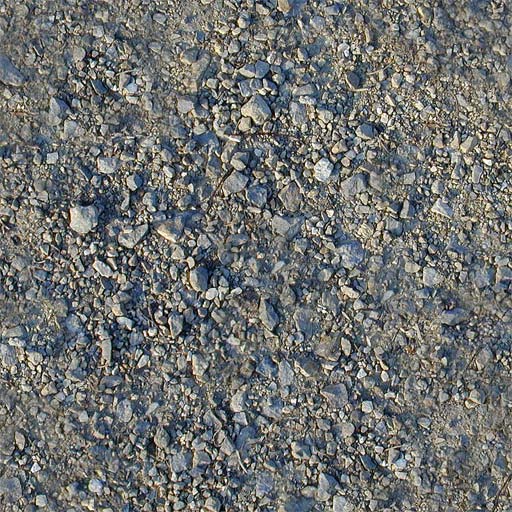

Supplement: S3 File — (ZIP) [file pone.0198788.s014.zip › farsa-1.4.5/worldsim/textures/ground/pebble03.jpg]

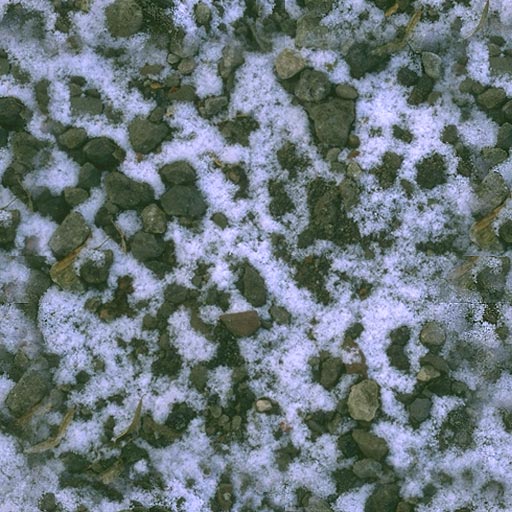

Supplement: S3 File — (ZIP) [file pone.0198788.s014.zip › farsa-1.4.5/worldsim/textures/ground/snowy01.jpg]

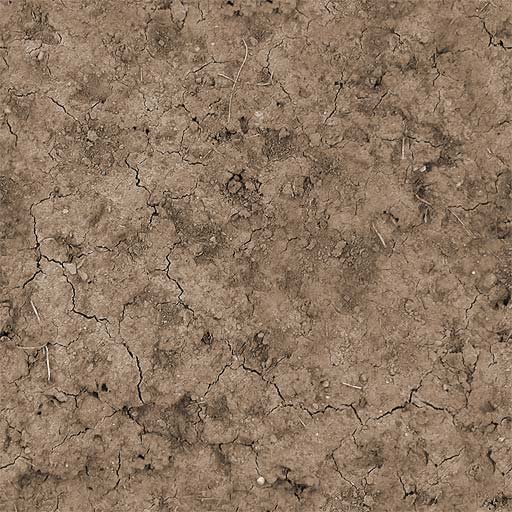

Supplement: S3 File — (ZIP) [file pone.0198788.s014.zip › farsa-1.4.5/worldsim/textures/ground/mud02.jpg]

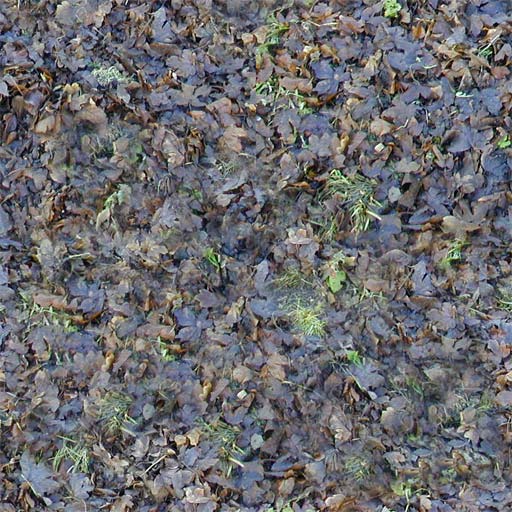

Supplement: S3 File — (ZIP) [file pone.0198788.s014.zip › farsa-1.4.5/worldsim/textures/ground/ground04.jpg]

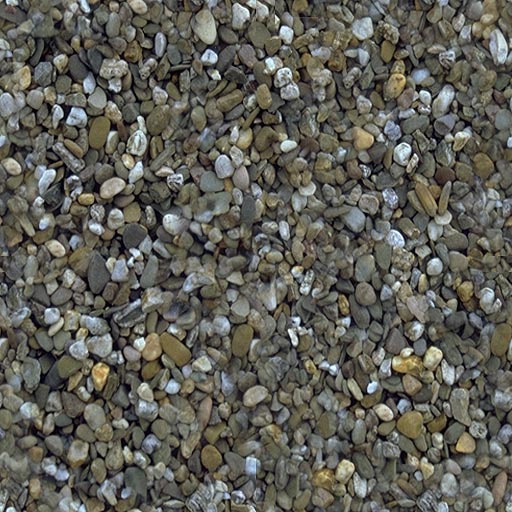

Supplement: S3 File — (ZIP) [file pone.0198788.s014.zip › farsa-1.4.5/worldsim/textures/ground/pebble01.jpg]

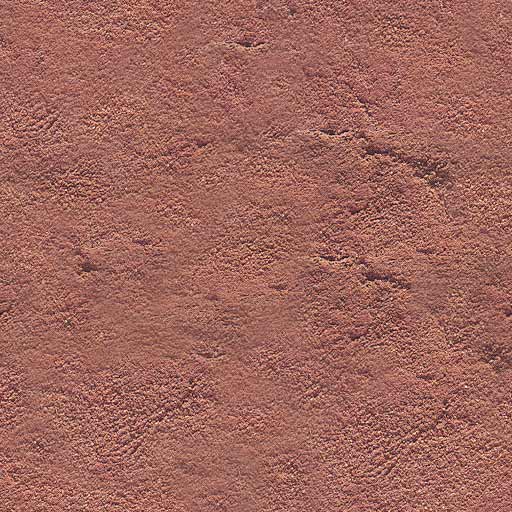

Supplement: S3 File — (ZIP) [file pone.0198788.s014.zip › farsa-1.4.5/worldsim/textures/ground/ground01.jpg]

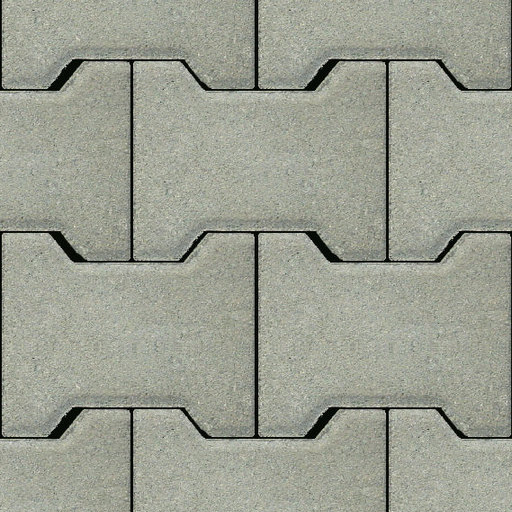

Supplement: S3 File — (ZIP) [file pone.0198788.s014.zip › farsa-1.4.5/worldsim/textures/ground/cobbles02.jpg]

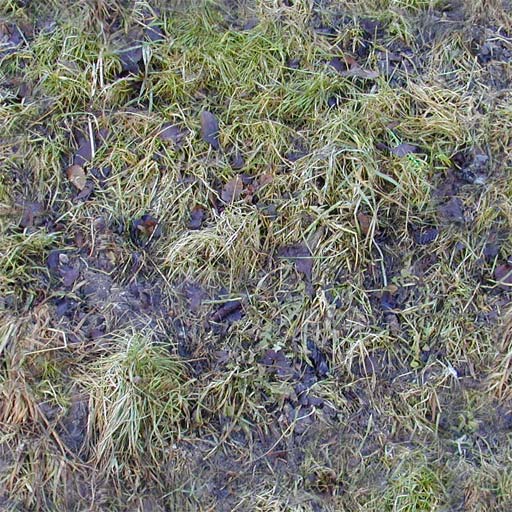

Supplement: S3 File — (ZIP) [file pone.0198788.s014.zip › farsa-1.4.5/worldsim/textures/ground/ground03.jpg]

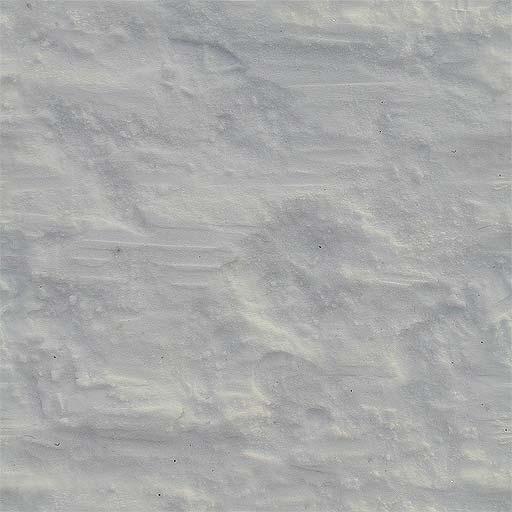

Supplement: S3 File — (ZIP) [file pone.0198788.s014.zip › farsa-1.4.5/worldsim/textures/ground/snowy02.jpg]

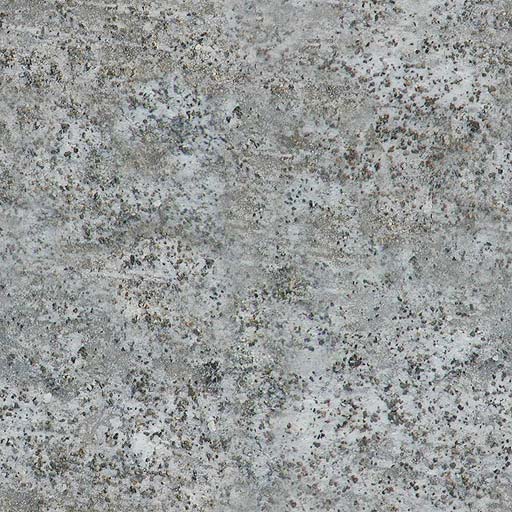

Supplement: S3 File — (ZIP) [file pone.0198788.s014.zip › farsa-1.4.5/worldsim/textures/ground/snowy04.jpg]

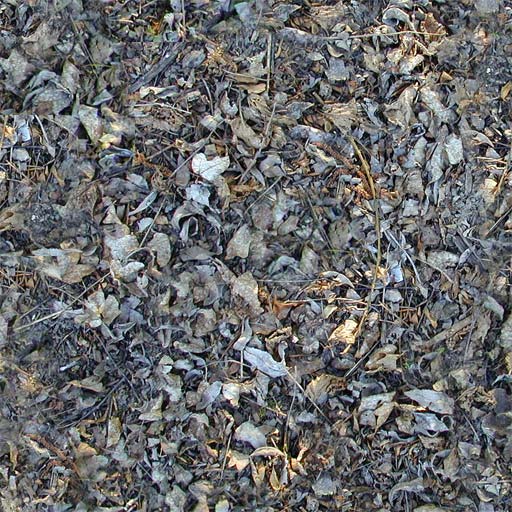

Supplement: S3 File — (ZIP) [file pone.0198788.s014.zip › farsa-1.4.5/worldsim/textures/ground/ground07.jpg]

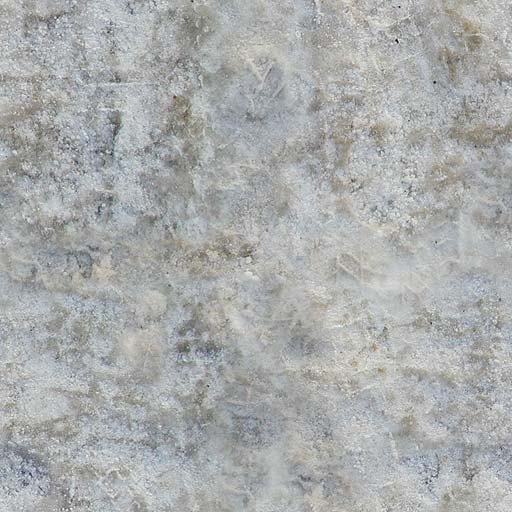

Supplement: S3 File — (ZIP) [file pone.0198788.s014.zip › farsa-1.4.5/worldsim/textures/ground/snowy05.jpg]

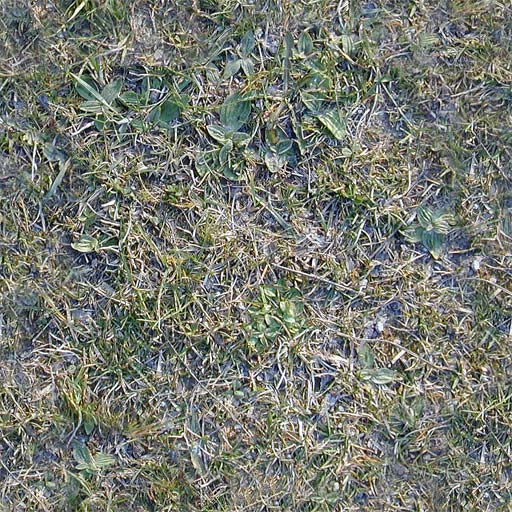

Supplement: S3 File — (ZIP) [file pone.0198788.s014.zip › farsa-1.4.5/worldsim/textures/ground/ground08.jpg]

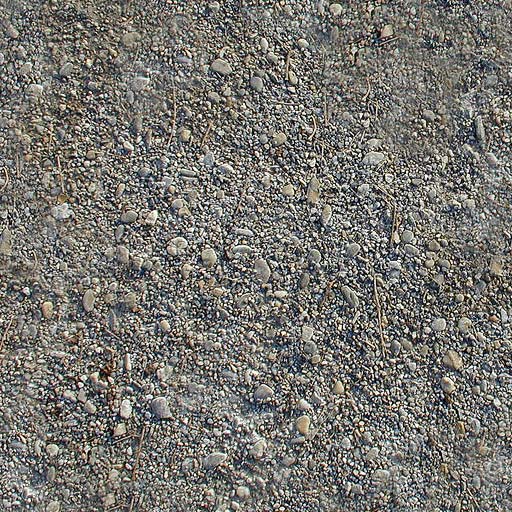

Supplement: S3 File — (ZIP) [file pone.0198788.s014.zip › farsa-1.4.5/worldsim/textures/ground/ground02.jpg]

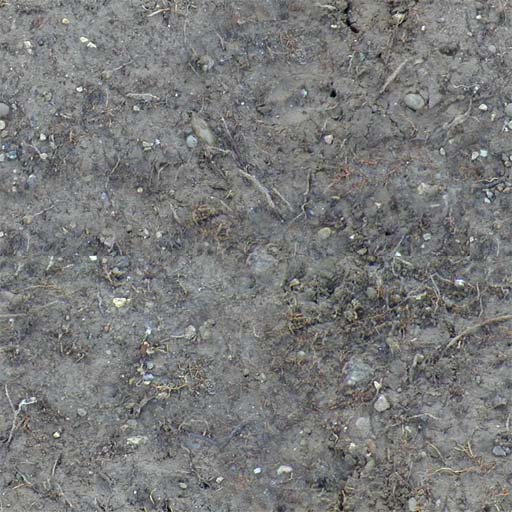

Supplement: S3 File — (ZIP) [file pone.0198788.s014.zip › farsa-1.4.5/worldsim/textures/ground/ground06.jpg]

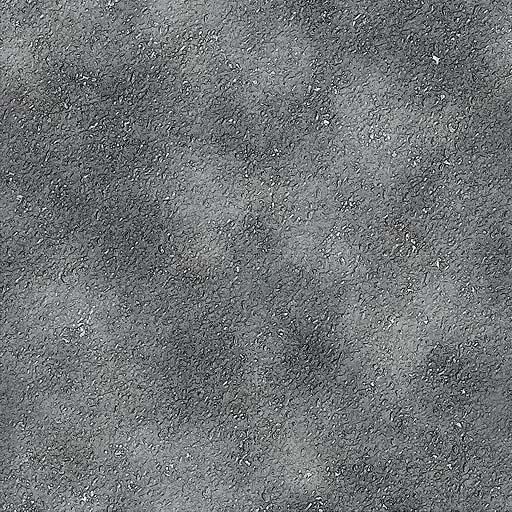

Supplement: S3 File — (ZIP) [file pone.0198788.s014.zip › farsa-1.4.5/worldsim/textures/ground/asph02.jpg]

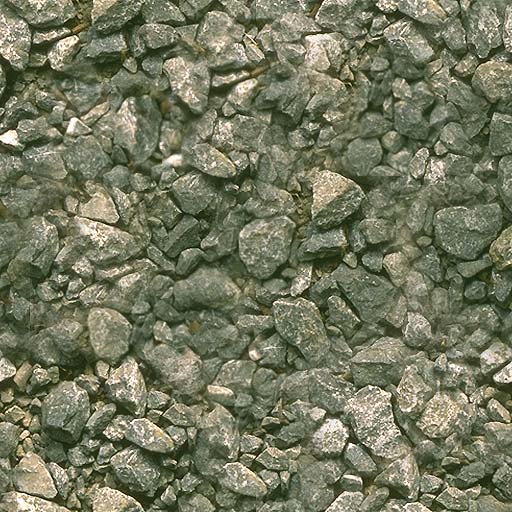

Supplement: S3 File — (ZIP) [file pone.0198788.s014.zip › farsa-1.4.5/worldsim/textures/ground/pebble02.jpg]
