# Supplementary material for: Maximizing adaptive power in neuroevolution
Source: PLoS One. 2018 Jul 18;13(7):e0198788. doi: 10.1371/journal.pone.0198788 (PMC6051599; doi:10.1371/journal.pone.0198788)
Supplement: S2 Table — Each number indicates the average performance obtained during 30 replications of the experiment. Generalization refers to the average performance obtained by post-evaluating the evolved networks on 1000 trials during which the initial states of the cart have been set randomly. The numbers in square brackets indicate the number of trials in which the agents manage to maintain the poles balanced for the entire duration of the trial during the post-evaluation test. The best performance for each condition is indicated in bold. (PDF) [file pone.0198788.s008.pdf]

**NEAT**	**FixedInitialStates**		**RandomlyVaryingInitialStates**	
**Double-Pole**	Add Neuron 1%	Add Neuron 10%	Add Neuron 1%	Add Neuron 10%
Population 20	0.770 [0.210]	0.503 [0.123]	0.115 [0.157]	0.112 [0.162]
Population 100	0.792 [0.274]	0.560 [0.181]	0.590 [0.676]	0.551 [0.621]
Population 500	0.817 [0.360]	0.601 [0.231]	0.642 [0.680]	0.615 [0.662]
Population 1000	**0.879 [0.397]**	0.605 [0.238]	**0.696 [0.710]**	0.681 [0.695]
**Delayed Double-Pole**				
Population 20	0.08 [0.025]	0.07 [0.017]	0.08 [0.021]	0.07 [0.015]
Population 100	0.12 [0.028]	0.24 [0.027]	0.12 [0.022]	0.235 [0.026]
Population 500	0.22 [0.031]	0.21 [0.030]	0.22 [0.030]	0.212 [0.031]
Population 1000	**0.292 [0.038]**	0.276 [0.036]	**0.30 [0.037]**	0.281 [0.035]
