# Supplementary material for: Maximizing adaptive power in neuroevolution
Source: PLoS One. 2018 Jul 18;13(7):e0198788. doi: 10.1371/journal.pone.0198788 (PMC6051599; doi:10.1371/journal.pone.0198788)
Supplement: S3 File — (ZIP) [file pone.0198788.s014.zip › farsa-1.4.5/docs/header.html]

$title


# $projectname - $projectnumber

Utilities Configuration
GA   
NNFW WorldSim
Total 99% Experiments
